# Abnormal Behavior in a Chromosome- Engineered Mouse Model for Human 15q11-13 Duplication Seen in Autism

**DOI:** 10.1016/j.cell.2009.04.024

**Published:** 2009-06-26

**Authors:** Jin Nakatani, Kota Tamada, Fumiyuki Hatanaka, Satoko Ise, Hisashi Ohta, Kiyoshi Inoue, Shozo Tomonaga, Yasuhito Watanabe, Yeun Jun Chung, Ruby Banerjee, Kazuya Iwamoto, Tadafumi Kato, Makoto Okazawa, Kenta Yamauchi, Koichi Tanda, Keizo Takao, Tsuyoshi Miyakawa, Allan Bradley, Toru Takumi

**Affiliations:** 1 Osaka Bioscience Institute, Suita, Osaka 565-0874, Japan; 2 Kyoto University Graduate School of Biostudies, Kyoto University Graduate School of Medicine, Sakyo, Kyoto 606-8501, Japan; 3 Department of Molecular Neuroscience, Kyoto University Graduate School of Medicine, Sakyo, Kyoto 606-8501, Japan; 4 Tsukuba Research Institute, Banyu Pharmaceutical Co. Ltd., Tsukuba, Ibaraki 300-2611, Japan; 5 The Wellcome Trust Sanger Institute, Hinxton, Cambridge CB10 1SA, UK; 6 Brain Science Institute, RIKEN, Wako, Saitama 351-0198, Japan; 7 Graduate School of Biomedical Sciences, Hiroshima University, Minami, Hiroshima 734-8553, Japan; 8 Frontier Technology Center, Kyoto University Graduate School of Medicine, Sakyo, Kyoto 606-8501, Japan; 9 Division of Systems Medicine, Institute for Comprehensive Medical Science, Fujita Health University, Toyoake, Aichi 470-1192, Japan

## Abstract

Substantial evidence suggests that chromosomal abnormalities contribute to the risk of autism. The duplication of human chromosome 15q11-13 is known to be the most frequent cytogenetic abnormality in autism. We have modeled this genetic change in mice by using chromosome engineering to generate a 6.3 Mb duplication of the conserved linkage group on mouse chromosome 7. Mice with a paternal duplication display poor social interaction, behavioral inflexibility, abnormal ultrasonic vocalizations, and correlates of anxiety. An increased MBII52 snoRNA within the duplicated region, affecting the serotonin 2c receptor (5-HT2cR), correlates with altered intracellular Ca^2+^ responses elicited by a 5-HT2cR agonist in neurons of mice with a paternal duplication. This chromosome-engineered mouse model for autism seems to replicate various aspects of human autistic phenotypes and validates the relevance of the human chromosome abnormality. This model will facilitate forward genetics of developmental brain disorders and serve as an invaluable tool for therapeutic development.

## Introduction

Autism is a common and heterogeneous neuropsychiatric disorder with manifestations of deficit in social interaction, impaired communication, and repetitive behavior or restricted interest (Volkmar and Pauls, 2003). Its definition has been extended to autism spectrum disorder (ASD), including autism-related disorders such as Asperger disorder and Rett's syndrome (DiCicco-Bloom et al., 2006; Geschwind and Levitt, 2007; Lord et al., 2000; Veenstra-VanderWeele et al., 2004). Autism is now considered a developmental brain disease (Belmonte et al., 2004; DiCicco-Bloom et al., 2006; Dykens et al., 2004; Geschwind and Levitt, 2007; Maestrini et al., 2000; Veenstra-VanderWeele and Cook, 2004; Vorstman et al., 2006). The first signs of autism appear at around 6 months, full diagnosis is usually made at 3 years, and symptoms usually persist throughout life. Autism is one of the most heritable neuropsychiatric disorders (Geschwind and Levitt, 2007), suggesting that genetic factors play an important role in its etiology (Vorstman et al., 2006). Candidate chromosomal regions and specific genes have been investigated (Belmonte et al., 2004; Folstein and Rosen-Sheidley, 2001; Persico and Bourgeron, 2006; Polleux and Lauder, 2004). There are likely to be de novo mutations, chromosomal abnormalities and common genetic variants that contribute to the genetic etiologies of autism (Abrahams and Geschwind, 2008; Geschwind, 2008; Geschwind and Levitt, 2007; Ramocki and Zoghbi, 2008). Several knockout mice have also been reported as “putative” autistic models, as judged from their phenotypes, but the molecular mechanism responsible for the pathophysiology of autism is far from complete.

Abnormalities of chromosomes are thought to account for 10% to 20% of autism cases (Beaudet, 2007). A recent study has established de novo germline mutations including copy number variants (CNVs) as a more significant risk factor for ASD than previously recognized (Sebat et al., 2007). Paternally or maternally inherited deletions of human chromosome 15q11-13 occur quite frequently, when they affect the imprinted region this is recognized as Prader-Willi syndrome or Angelman syndrome, respectively (Nicholls and Knepper, 2001). Duplication of the same region is the only recurrent cytogenetic aberration associated with autism, occurring in up to 5% of autism cases (Belmonte et al., 2004; Bolton et al., 2004; Cook and Scherer, 2008; Dykens et al., 2004; Folstein and Rosen-Sheidley, 2001; Lord et al., 2000; Maestrini et al., 2000; Veenstra-VanderWeele et al., 2004; Veenstra-VanderWeele and Cook, 2004; Vorstman et al., 2006).

On the basis of conserved human/mouse linkage, we have generated mice with a 6.3 Mb duplication of mouse chromosome 7 mirroring the human chromosome 15q11-13 duplication. This mouse model displays several phenotypes that recapitulate aspects of the human condition and provides mechanistic insight into the disease.

## Results

### Construction of a 6.3 Mb Duplication on Mouse Chromosome 7

**Figure 1:**
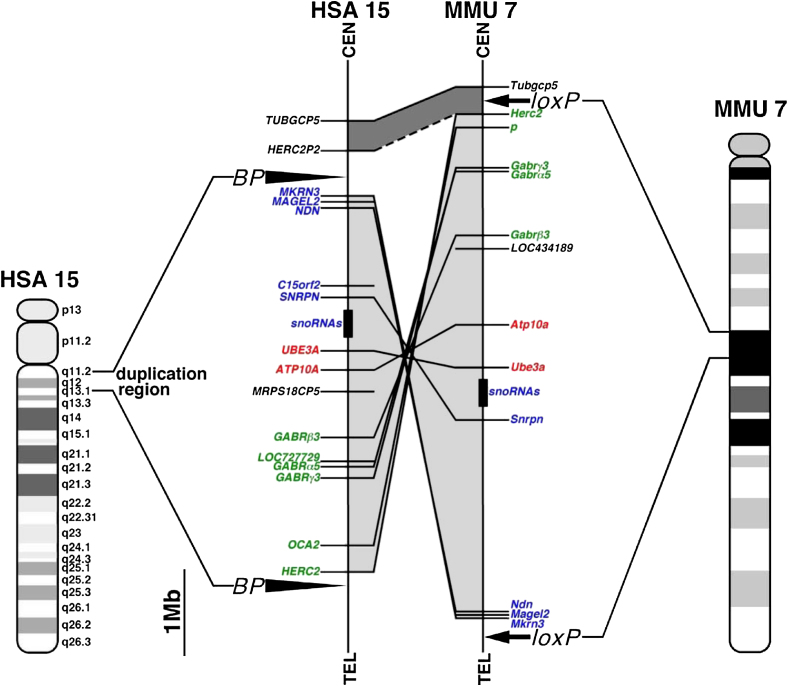
Human Chromosome 15q11-13 and Mouse Chromosome 7 Schematic representation of the genomic regions in the human and mouse genomes. Details of conserved linkage in human 15q11-13 and mouse chromosome 7 are shown. The paternally, maternally expressed, and nonimprinting genes were labeled with blue, red, and green, respectively. The two arrowheads (BP) represent the common breakpoints, and the two arrows represent the targeting sites of 2 loxP sequences. Genomic segments that show linkage conservation (i.e., identical gene order) in humans and mice are connected by dark shading if the gene orders are in the same direction relative to their respective centromeres. If the gene orders in the syntenic segments are in opposite orientations, they are connected by light shading.

**Figure 2:**
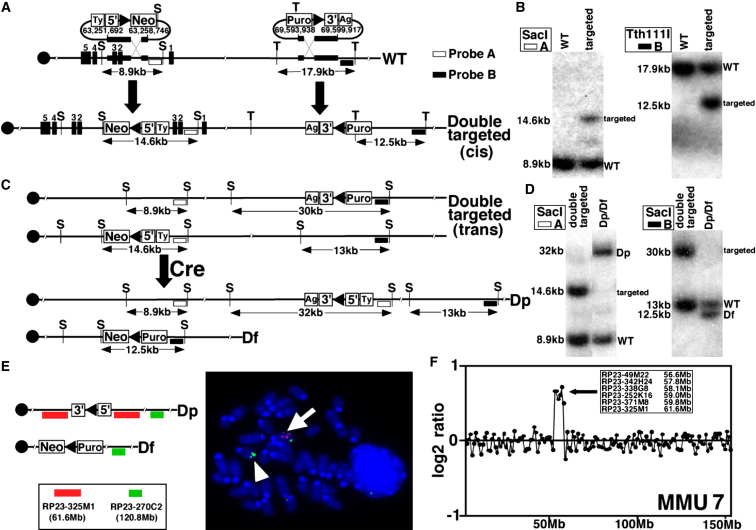
Engineering an Interstitial Duplication on Chromosome 7 (A) Insertional double targeting and genomic coordinates, NCBI build m37. S, SacI; T, Tth111I. (B) Southern blot analysis of ES cell DNA samples. (C) Cre/*loxP* recombination generates duplication and deletion chromosomes. (D) Southern blot analysis of ES cell DNA confirming the duplication. (E) Confirmation by FISH. The probes used are shown on the left. The red and green bars represent the probes located within and outside the duplicated region, respectively. The white arrow and arrowhead represent the *Dp* and *Df* alleles, respectively. (F) A BAC array-CGH profile of chromosome 7 from mice with the duplication. Log2-transformed hybridization ratios of duplicated mouse DNA versus WT DNA are plotted.

Human chromosome 15q11-13 has a conserved linkage group on mouse chromosome 7 (Figure 1). Chromosomal engineering (van der Weyden and Bradley, 2006) was used to construct an interstitial duplication of mouse chromosome 7 corresponding to the region between common breakpoints in human chromosome 15q11-13. Sequential rounds of insertional gene targeting were used to insert the selection cassettes and *loxP* sites required for chromosome engineering proximal to the *Herc2* and distal to *Mkrn3* (Figures 1 and 2A). A double-targeted clone in which the targeting had occurred on the homologous chromosomes (*trans*) was transiently transfected with a Cre expression plasmid to induce recombination between the *loxP* sites, which generated clones with a balanced duplication (*Dp*) and deletion (*Df*) (Figure 2C). Recombinants were recovered by hypoxanthine-aminopterin-thymidine (HAT) selection at a frequency of 26 × 10^−7^ per electroporated cell and confirmed via Southern blot analysis (Figures 2B and 2D) and fluorescence in situ hybridization (FISH) (Figure 2E). The deletion and duplication alleles were transmitted and established in the germ line by standard procedures, and the expected increase in genomic copy number of this region was confirmed with comparative genomic hybridization (CGH) on a mouse bacterial artificial chromosome (BAC) microarray (Figure 2F).

### Increased Gene Expression of Duplicated Genes

The duplication allele was transmitted at normal Mendelian ratios from both females and males. Mice with a maternally (*matDp/+*) and paternally (*patDp/+*) inherited duplication bred normally and were fertile. The *patDp/+* male mice began to show an increase in body weight compared to wild-type (WT) mice after 15 weeks, and the body weight of *patDp/+* was significantly greater than that of the WT after 20 weeks (data not shown).

We performed histological analyses of the adult brain as well as that of brains at postnatal day 0 (P0), P7, and P14 to screen for morphological changes. No significant abnormality was detected in H&E-stained sections of the olfactory bulb, cerebral cortex, hippocampus, amygdala, corpus callosum, and cerebellum either macroscopically or at the microscopic level (Figure S1 available online). The number of Purkinje cells in the cerebellum was not significantly different between mice with the duplication and WT mice (Figure S2). Bodian staining was also performed, and this did not reveal any significant abnormality in the cortex, hippocampus, amygdala, and cerebellum (data not shown).

**Figure 3:**
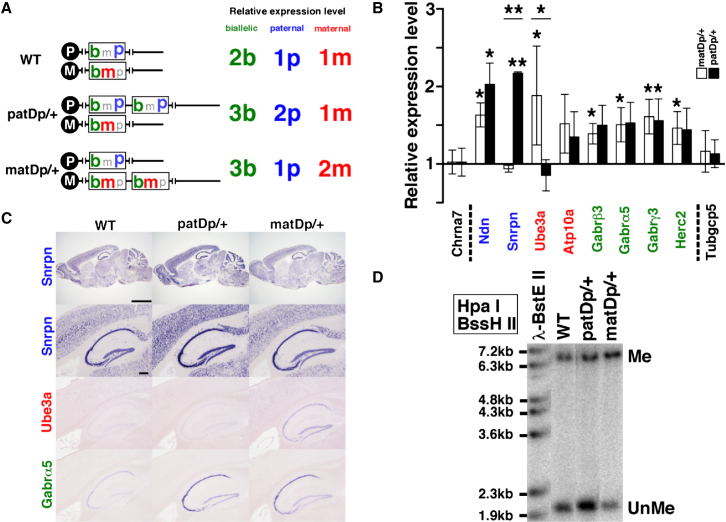
Gene Expression in Mice with the Duplication (A) Expected gene expression levels in wild-type (WT), paternal duplication (*patDp*/+), and maternal duplication (*matDp*/+) mice. (B) mRNA expression in the mouse adult brain of the listed genes analyzed by quantitative RT-PCR. The relative expression levels of *patDp*/+ (n = 4) and *matDp*/+ (n = 4) were compared with WT (n = 7) normalized to 1.0. Blue, red, and green indicate paternally expressed, maternally expressed, and nonimprinted genes, respectively. Dotted lines show the boundaries of the chromosomal rearrangement. Error bars represent the standard error of the mean (SEM). ^∗∗^p < 0.0001, ^∗^p < 0.05. (C) *Snrpn*, *Ube3a*, and *Gabrα5* mRNA expression in the adult mouse brain (top row) and hippocampus (other rows) detected by in situ hybridization. Scale bars represent 2 mm (top row) and 200 μm (other rows). (D) Methylation analysis by Southern blotting. Me, methylated; UnMe, unmethylated.

The 6.3 Mb duplication includes the region of genomic imprinting. The relative expression levels of genes in the duplicated region are expected to vary depending on whether they are imprinted and on their mode of inheritance (Figure 3A). Therefore, gene expression was assessed by quantitative RT-PCR in the brains from *patDp/+* and *matDp/+* mice, and the results were normalized to those in WT mice (Figure 3B). In the adult brain, *Ndn*, *Snrpn*, *Ube3a*, *Gabrβ3*, *Gabrα5*, and *Herc2* genes in the duplicated region were highly expressed, whereas the expression of others was relatively less abundant. The messenger RNA (mRNA) levels of the paternally expressed genes, *Ndn*, and *Snrpn*, were increased more than 2-fold in the *patDp/+* mice. Unexpectedly, *Ndn* also exhibited increased expression levels in *matDp/+*, though the levels were lower, while *Snrpn* showed the expected level (no change). The maternally expressed gene, *Ube3a*, showed an approximately 2-fold increase in *matDp/+* mice. The expression of *Atp10a* did not show any significant difference between *patDp/+* and *matDp/+* mice. The nonimprinting genes, GABA_A_ receptor subunits and *Herc2*, showed the expected 1.5-fold increase in mice with the duplication. *Chrna7* and *Tubgcp5*, both of which are located outside the duplicated region, did not show any significant change in their expression levels. In other tissues, the expression phenotypes reflected those in the brain (Figure S3). The expression of each gene in various areas of the adult mouse brains was examined by in situ hybridization (Figure 3C). Alterations in the expression patterns of these genes in *patDp/+* and *matDp/+* brains were unremarkable, although the expression level of *Snrpn* in the hippocampus seemed to be higher in the *patDp*/+ mice, whereas that of *Ube3a* seemed to be higher in the *matDp*/+ mice.

DNA methylation is an epigenetic modification in imprinted regions and is found in 15q11-13 (Nicholls and Knepper, 2001). The imprinting center (IC) is localized to the 5′ end of *Snurf-Snrpn* locus. This region is methylated (Me) or unmethylated (UnMe) in maternal and paternal alleles, respectively. We thus examined allele-specific methylation by Southern blotting with a *Snurf* probe by using methylation-sensitive (BssHII) and -insensitive (HpaI) restriction enzymes. The ratios of Me and UnMe bands were 1:2 and 2:1 in *patDp/+* and *matDp/+* mice, respectively, in contrast to 1:1 in the WT (Figure 3D). These results suggest that allele-specific methylation is conserved in the mice with the duplicated allele.

### *patDp/+* Mice Display Social Abnormalities

To analyze the effect of the chromosomal duplication on behavior, we performed a comprehensive battery of behavioral tests (Crawley, 2007; Takao et al., 2007; Yamasaki et al., 2008). We observed significant differences between the WT and mice with a duplication in the several tests described below (Tables S1 and S2). The diagnosis of autism is based on behavioral criteria (Volkmar and Pauls, 2003). Therefore, a valid mouse model should reflect behavioral symptoms, including impairment in social interaction (Crawley, 2004; Moy et al., 2006).

**Figure 4:**
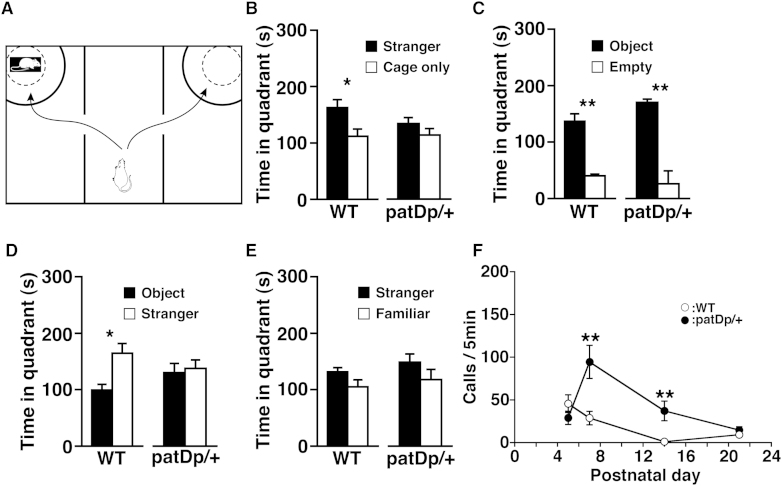
*patDp*/+ Mice Show Social Abnormalities (A–E) Three-chamber test. (A) Schematic representation of the three-chambered apparatus. The quadrant-like spaces between the full and dotted lines were used for quantitative analysis. (B) A stranger mouse was restricted in one of the side chambers in a wire cage, and only an empty wire cage was placed in the opposite chamber. Comparison of time spent in the quadrant spaces between “Stranger” and “Cage” for WT (n = 14) and *patDp*/+ mice (n = 13) is shown. Error bars represent the SEM. ^∗^p < 0.05. (C) A novel object A (a dodecahedral pole) was placed in a cage in the chamber on one side, and no object in the chamber on the other side. Both WT and *patDp/+* mice spent more time around the cage with a novel object. n = 11. ^∗∗^p < 0.001. (D) Another novel object B (a cone) was placed in a cage in the chamber on one side, and an adult conspecific mouse (C57BL/6J) that has had no previous contact with the subject (test mouse) in a cage in the chamber on the other side. WT mice spent more time around the stranger mouse. n = 11. ^∗^p < 0.05. (E) A novel stranger mouse (C57BL/6J) is placed in a cage in the chamber on one side and a familiar mouse that was used in a previous test in D is placed in the chamber on the other side. n = 11. These data were evaluated by the t test. (F) Maternal separation-induced ultrasonic vocalizations at P5, P7, P14, and P21 (or P22). n = 32, 40, 40, and 16, respectively for *patDp*/+; n = 24, 39, 39, and 12, respectively for the WT. ^∗∗^p < 0.005. For (B)–(F) error bars represent the SEM.

A three-chamber social interaction test (Crawley, 2004; Nadler et al., 2004) was performed (Figure 4A). The mouse to be tested was placed in the central chamber and could move freely among the three chambers. A stranger mouse was placed in one of the side chambers in a wire cage, and only a cage was placed in the opposite chamber. WT mice tended to contact the stranger mouse, and the time spent with the stranger mouse in the quadrant location depicted by the line in Figure 4A was significantly higher than the time spent in the corresponding location in the opposite chamber with the empty cage (Figure 4B). In contrast, the *patDp/+* mice exhibited no significant difference in time spent between the quadrant spaces of either side (Figure 4B). These phenotypes were also observed in mice with a different background under similar experimental conditions (Figure S4). To confirm that these results are due to specific changes in social behavior, we further performed the three-chamber test under different conditions. First, we assessed the reaction of mice to a novel inanimate object. Both *patDp/+* and WT mice spent more time around the cage with a novel inanimate object compared with the empty cage, and no significant difference between *patDp/+* and WT mice was observed (Figures 4C and S5A). Second, the simultaneous interactions with a novel mouse and another novel object were compared. WT mice spent more time around the cage with a novel mouse than with a novel object, whereas *patDp/+* mice showed no significant difference in time spent around the cages with a novel mouse and object (Figures 4D and S5B). Third, the interactions of the mice with a novel and familiar mouse were also compared. In WT mice, although the time spent around the cages was not significantly altered, the number of entries around the novel mouse tended to be greater than that of the familiar mouse (p = 0.0541), whereas in *patDp/+* mice, no significant difference between the novel and familiar mouse was found (Figure 4E and S5C). These results suggest that WT mice are more interested in a novel mouse than a novel inanimate object, but that *patDp/+* mice have decreased sociability compared with the WT, which may be analogous to the impairment in appropriate social interaction often seen in autistic patients (Crawley, 2004). On the other hand, *matDp/+* mice were indistinguishable from WT mice (Figure S6). Since social behavior in mice is to a large extent olfactory driven, we examined the olfactory system of the mice anatomically (by immunohistochemistry) and functionally (by olfactory habituation/dishabituation test), and we excluded any defects of the olfactory system in *patDp/+* mice (data not shown).

**Figure 5:**
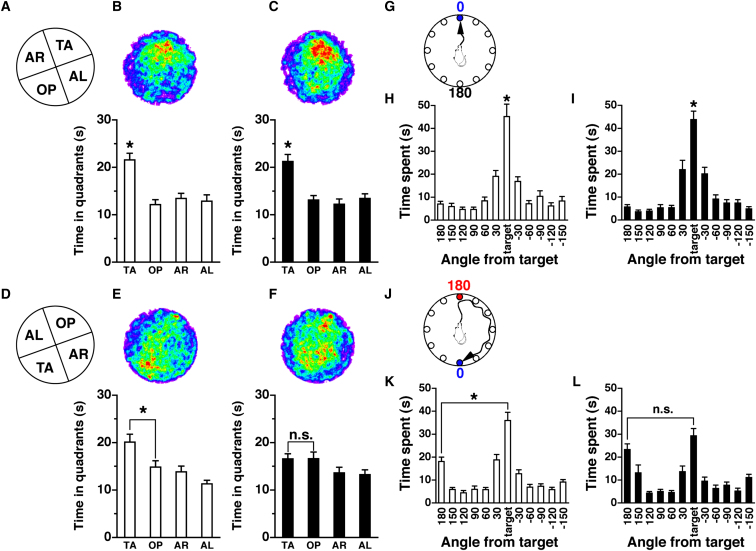
*patDp/+* Mice Show Behavioral Inflexibility in the Morris Water Maze and Barnes Maze Tests (A–F) Morris water maze test; n = 20 for both genotypes. White bar, WT; black bar, *patDp/+*. (A) The configuration of the four quadrants in the probe test after the original training (TA, target quadrant; OP, opposite quadrant; AR, adjacent right quadrant; AL, adjacent left quadrant). (B and C) Probe test after the original training. Upper panels indicate averaged swimming traces of the swim pattern for WT (B) and *patDp/+* (C) mice. Warmer color represents more time spent. Lower panels show the quadrant occupancy for WT (B) and *patDp/+* (C) mice. Both WT and *patDp/+* mice showed significantly more time spent in the target quadrant compared with the other quadrants [WT, *F*(3,76) = 12.86, p < 0.0001; *patDp/+*, *F*(3,76) = 13.31, p < 0.0001; Newman-Keuls post hoc comparison (trained quadrant more than all the other quadrants); p < 0.01 for both genotypes]. (D) The configuration of the four quadrants in the reversal probe test. (E and F) Reversal probe test. Upper panels indicate averaged swimming traces of the swim pattern for WT (E) and *patDp/+* (F) mice. Lower panels show the quadrant occupancy for WT (E) and *patDp/+* (F) mice. While WT mice spent significantly more time in the reversed target quadrant, *patDp/+* mice showed no significant difference in the time spent between the quadrants [WT, *F*(3,76) = 8.20, p < 0.0001; *patDp/+*, *F*(3,76) = 2.40, p = 0.0745; Neuman-Keuls post hoc comparison (trained quadrant more than all the other quadrants); WT, p < 0.01; *patDp/+*, p > 0.05]. (G–L) Barnes maze test, n = 22 for both genotypes. White bar, WT; black bar, *patDp/+*. (G) The target position in the Barnes maze original probe test. The hole at 0 degrees is the correct hole chosen as the target. (H and I) Both genotypes could learn the target position spatially in the original probe test [WT, *F*(11,252) = 25.47, p < 0.0001; *patDp/+*, *F*(11,252) = 32.27, p < 0.0001; Bonferroni post hoc comparison (time spent around the target position more than those of all the other holes), both genotypes, p < 0.01]. (J) The target position in the Barnes maze reversal probe test. The target at 0 degrees is moved to the opposite position. The original target position is labeled in red, at 180 degrees, and the new target position is labeled in blue, at 0 degrees. (K and L) While WT mice could learn the new target position flexibly, *patDp/+* mice could not respond as flexibly as WT mice [WT, *F*(11,252) = 29,08, p < 0.0001; *patDp/+*, *F*(11,252) = 16.04, p < 0.0001; Bonferroni post hoc comparison (target versus 180 degrees), WT, p < 0.01; patDp/+, p > 0.05]. ^∗^p < 0.01; n.s., not significant (p > 0.05). Furthermore, time spent around the 180 degree position and its neighboring 150 degree position was increased in *patDp/+* mice compared to the WT (180 degrees, p < 0.1; 150 degrees, p < 0.05). For (B), (C), (E), (F), (H), (I), (K), and (L), error bars represent the SEM.

To measure behavioral flexibility, we habitually trained mice and then analyzed their responses to a change in routine in a reversal task by using the Morris water maze test and the Barnes maze test, which have been generally validated for spatial learning and memory (Crawley, 2007; Miyakawa et al., 2001). The Morris water maze is a spatial navigation task in which the mouse swims to find a hidden platform. Mice were trained to locate the correct platform to escape from the water. Both *patDp/+* and WT mice learned the target quadrant (TA in Figures 5A–5C), suggesting that there was no impairment in spatial learning in *patDp/+* mice. When the target platform was then moved to the opposite area (TA in Figure 5D), WT mice spent significantly more time in the new TA quadrant compared with the opposite quadrant (OP) (Figure 5E). On the other hand, *patDp/+* mice exhibited no difference between time in the TA and OP (Figure 5F).

The Barnes maze is a circular white platform with 12 holes (Figures 5G and 5J). One of the holes exits into a dark box called the target initially placed at 0 degrees (Figure 5G). Mice were trained to locate the correct hole to exit into the escape box. Both *patDp/+* and WT mice learned to identify the target at the 0 degree point, and there was no observable difference between the two mice (Figures 5H and 5I), again suggesting that there was no impairment in spatial learning of *patDp/+* mice. When the target was moved to the opposite side (Figure 5J), both *patDp/+* and WT mice could find the target; however, compared with the WT, *patDp/+* mice stayed less in the new target position and more in the 180 or ±150 degree position, which is the position of the original target or in its direction (Figure 5L). The time spent between the target and the 180 degree position was significantly different in WT mice (Figure 5K), whereas there was no significant difference in *patDp/+* mice (Figure 5L). The Barnes maze test was also performed in mice with a different background, and the results were consistent (Figures S7A and S7B). Conversely, *matDp/+* mice did not show any significant change even in reversal learning compared with WT mice (Figures S8A and S8B). These results suggest that *patDp/+* mice do not respond as flexibly as WT and *matDp/+* mice to a change in situation, which may be comparable to the inflexibility in routine that is characteristic of autism (Crawley, 2004), although we should acknowledge that it is far from clear how cognitive deficits in reversal learning are related to the behavioral deficits in autism even in humans and even less clear from mouse to humans (Geurts et al., 2009). Furthermore, we found the lack of reversal deficits in the T maze test (data not shown). Perhaps reversal deficits are only apparent during aversively motivated escape behaviors and not appetitively motivated approach behaviors.

To see impairment in communicative behavior, we measured ultrasonic vocalizations (USVs) of neonatal mice that were separated from their dams (Figure 4F). These USVs are thought to be distress signals (Crawley, 2007) and may be related to communication between a dam and her pups (Crawley, 2004). In WT mice, the USVs have a normal developmental course, emerging soon after birth, peaking at around P5, and then decreasing to almost zero at around P14, the time of eye opening when the development of alternative communication may occur (Noirot, 1966). The USVs emitted by *patDp/+* pups at P7 and P14 were markedly greater than those of WT pups (Figure 4F). In the *patDp/+* pups, the peak in the numbers of USVs seemed to be delayed, and the USVs were still present at P14, when those of WT mice had disappeared, suggesting that *patDp*/+ mice may be developmentally abnormal in comparison with the WT. Detailed frequency analysis revealed that most of the USVs at P7 and P14 emitted by *patDp*/+ pups were mainly in the 50–70 kHz frequency range, with some over 70 kHz, the latter of which was not seen in WT pups (Figure S9). No difference in USVs was observed between *matDp*/+ and WT pups (Figure S8C), suggesting that communicative development between dam and neonatal mice in *patDp*/+ is different from that in WT or *matDp*/+ pups. This larger number of USVs in *patDp*/+ pups may reflect higher anxiety and fear in response to stress (Crawley, 2007).

We therefore examined vocalizations in adult animals, where the effects of anxiety and novel environments may be more controlled. Since adult mice emitted both audible and ultrasonic vocalizations, we measured vocalizations consisting of both frequencies. In a resident-intruder paradigm, the total number of vocalizations ranging from both audible and ultrasonic bands emitted by pairs of *patDp/+* mice was significantly decreased compared with that of a WT pair (Figure S10). The vocalizations ranging in the ultrasonic bands in pairs of *patDp/+* mice also tended to be lower than those in pairs of WT mice. The behavior between resident and intruder mice in each genotype was indistinguishable between the genotypes. These results suggest that vocal communication between pairs of *patDp/+* mice is decreased compared with a WT pair.

We observed the fear-related behaviors of *patDp/+* mice by conducting another test, the cued and contextual conditioning task, by using a 60 dB white noise tone and a mild foot shock (Figures S11A–S11C). No significant difference in the freezing rate between *patDp/+* and WT mice was seen during conditioning training (Figure S11A) or in the same contextual environment after 24 hr (Figure S11B). However, *patDp/+* mice showed higher freezing scores in the altered contextual environment than did the WT controls, especially during the first 3 min in the absence of the cue (Figure S11C). *matDp/+* mice displayed no difference from WT mice (Figures S12A–S12C). These results suggest that the *patDp/+* mice show a generalized fear. Additionally, we conducted the elevated plus maze test to examine anxiety (Figures S11D–S11G). As compared with the WT, the *patDp/+* mice showed no significant difference in distance traveled (Figure S11D), whereas the number of entries into the arms and time in the open arms were significantly decreased in the *patDp/+* mice (Figures S11E and S11F), but not in the *matDp/+* mice (Figures S12D–S12G). These results suggest that the *patDp/+* mice show increased anxiety, a feature common in autistic individuals (Crawley, 2004).

### 5-HT2c Receptor Signaling Is Altered in *patDp*/+ Neurons

**Figure 6:**
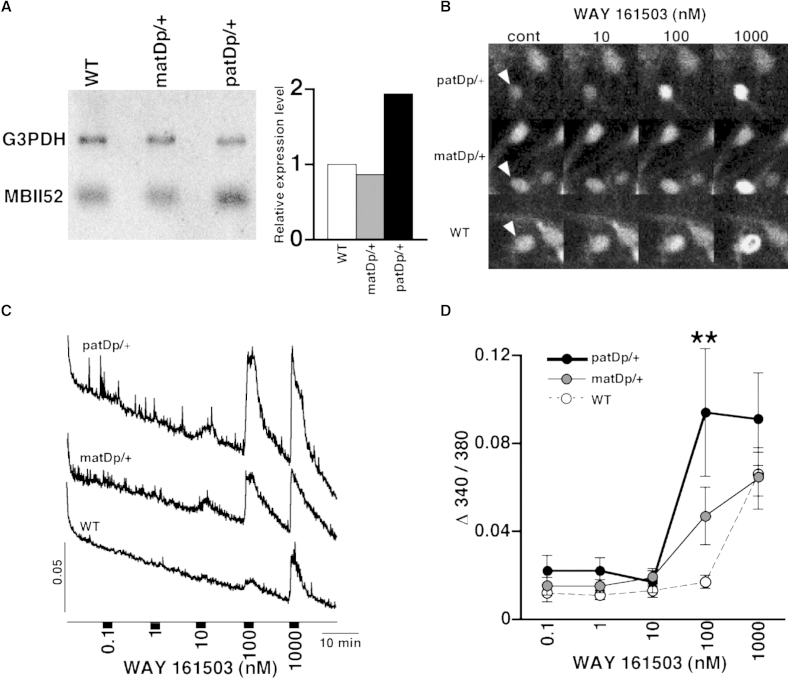
[Ca^2+^]_i_ Response by a 5-HT2cR Agonist in Neurons (A) Northern blot analysis of MBII52. Quantitative data are shown in the right panel, where MBII52 expression in WT is defined as 1. (B–D) The effect of WAY 161503 on [Ca^2+^]_i_ in primary cultured neurons. Representative images (responding cells are indicated by an arrowhead) and average responses under various concentrations of agonist are shown in (B) and (C), respectively. Averaged data for the concentration-dependent effect of WAY 161503 are indicated in (D). Error bars represent the SEM. n = 17 for *patDp*/+, n = 15 for *matDp*/+, n = 18 for WT. ^∗∗^p < 0.001.

Our behavioral tests demonstrated that *patDp*/+ mice show abnormal behaviors. The primary benefit of the model mouse system is that it allows us to study abnormality at the molecular level. To demonstrate this possibility, we examined one molecular candidate, i.e., a small nucleolar RNA (snoRNA). It has been reported that a brain-specific snoRNA, HBII52, the human ortholog of MBII52, plays a role in posttranscriptional modification of the serotonin 2c receptor (5-HT2cR), a G protein-coupled receptor (GPCR) (Kishore and Stamm, 2006), which may cause amino acid substitutions in the second intracellular domain of this receptor. The 5-HT2cR is the only GPCR that has been shown to undergo physiologically important editing of its pre-mRNA by adenosine deamination (A-to-I editing), resulting in amino acid substitutions (Seeburg, 2002). We examined MBII52 RNA expression in the brain by RNA blot hybridization (Figure 6A). Since the locus including MBII52 is maternally imprinted, the expression of MBII52 in the *patDp*/+ mouse brains was approximately twice as much as that in the WT or *matDp*/+ brains. We next analyzed the editing ratio of 5-HT2cR RNA at five potential sites that are located in the second intracellular domain. RNA editing ratios in *patDp*/+ at the A and B sites were significantly higher than those in the WT, and *patDp*/+ editing ratios at the D site were higher than *matDp*/+, whereas no significant difference was found for the editing frequency at the E and C sites among the three types of mice (Figure S13).

Because 5-HT2cR induces an increase in the intracellular calcium level ([Ca^2+^]_i_) via G proteins coupled to phospholipase C, we asked whether altered amounts of MBII52 would affect the [Ca^2+^]_i_ response via altered coupling efficiency between 5-HT2cR and G proteins. To analyze the serotonergic signals in neurons derived from mouse brains, we examined the effects of 5-HT2cR on [Ca^2+^]_i_ in primary cultured neurons by using microspectrofluorimetric techniques and the fluorescent indicator Fura-2 (Figure 6B). A specific agonist for 5-HT2cR, WAY 161503, induced an increase in [Ca^2+^]_i_. The response to 100 nM WAY 161503 in *patDp*/+ neurons was significantly higher than that in the WT (Figures 6C and 6D). These results demonstrate that substantial alterations in the amount of MBII52 RNA of the *patDp*/+ mice resulted in a significantly increased [Ca^2+^]_i_ response to 5-HT2cR signaling, suggesting that this alteration in serotonergic signaling may contribute to the abnormal behavior seen in the *patDp*/+ mice.

## Discussion

Ideal animal models of human neuropsychiatric disorders should not only phenocopy relevant human symptoms, but the phenotypes should also be based on similar underlying mechanisms acting both physiologically and genetically (Crawley, 2004). Several kinds of animal models for autism have been reported (Moy et al., 2006; Murcia et al., 2005; Persico and Bourgeron, 2006). Knockouts or knockin of single candidate genes, such as genes in the oxytocin-vasopressin system, dishevelled-1 (*Dv1*), engrailed2 (*En2*), *Pten*, and neuroligins have been reported as possible autistic model mice (DiCicco-Bloom et al., 2006; Jamain et al., 2008; Kwon et al., 2006; Lijam et al., 1997; Moretti et al., 2005; Tabuchi et al., 2007; Winslow and Insel, 2002; Young, 2007). Ours mirrors a chromosomal abnormality found in human autistic patients. In this regard, the chromosome-engineered mouse described here is a model mouse for autism that parallels both phenotypic and genotypic aspects of the human disease.

In the rotarod test, *patDp*/+ mice exhibited a significantly greater improvement of rotarod performance than did WT mice (Figure S14). This result may simply mean that *patDp*/+ mice possess higher motor coordination/learning ability compared with WT mice, but taken together with the results of the reversal learning, it can be interpreted that *patDp*/+ show better stereotypic behavior. This motor stereotypy or better performance in repetitive tests of motor coordination has also been reported in other models (Caston et al., 1998; Kwon et al., 2006). In addition to the major symptoms, there are several associated manifestations of emotional behavior in autism, such as anxiety, fear, and depression. Indeed *patDp/+* mice displayed these signs in the cued and contextual conditioning fear test, the elevated plus maze test, and the Porsolt forced swim test (Figures S11 and S15). Mao et al. reported that a patient with paternal duplicated 15q11-13 displayed depression and anxiety in addition to significant behavioral problems and obesity (Mao et al., 2000). Furthermore, in the eight-arm radial maze test, we noticed strange behavior in *patDp/+* mice. Even after training with dietary restriction, several mice did not seem to be eager to eat food. In addition, the latency of *patDp/+* mice to approach food was significantly longer than that of WT mice in the T maze test. These findings might reflect the increase in latency to feed observed in the novelty-suppressed feeding (NSF) test (Santarelli et al., 2003). These behavioral phenotypes may imply that *patDp/+* mice have greater fear and tend to freeze in novel environments or have a lack of desire.

In this study, mice with a paternal duplication showed abnormal phenotypes compared with WT mice. Reports on human autism associated with a paternal duplication have been accumulating (Bolton et al., 2004; Mao et al., 2000; Mohandas et al., 1999; Roberts et al., 2002; Veltman et al., 2005), although it has also been reported that maternal duplication of 15q11-13 causes autism in humans (Cook et al., 1997). Provided that autistic patients with the chromosome 15q11-13 duplications are the small affected cases compared with overall autistic patients, one should re-evaluate more clinical cases with the use of currently available high-resolution genome analysis techniques such as array CGH, as well as multiple oligonucleotide array platforms (Lee and Lupski, 2006). Some epigenetic controls may be different between human and mouse. Although the methylation status revealed by analyzing one probe around the IC region in this study seems to be conserved also in the mouse, methylation in other regions remains unknown. Epigenetic, developmental, and environmental influences may affect marked variability in phenotypic expression (Veltman et al., 2005).

The link between social behaviors in rodents and social behavior in humans is difficult to establish. Our model would provide a powerful tool to explore its mechanism. It has been reported that serotonin may be involved in the pathophysiology of autism, because serotonin plays a role as a growth factor in the immature brain (Bonnin et al., 2007; Riccio et al., 2008). Increased serotonergic activity during development could damage the neurocircuitry involved in emotional responses to social stress and may have relevance to the symptoms of autism (Whitaker-Azmitia, 2005). The 5-HT2cR studied here, mapped to the X chromosome, may be a candidate molecule for human genetic studies of autism, and its ligand may be a potential lead for therapeutic targets. Another intriguing hypothesis is imbalance between excitatory and inhibitory neural signals at the developmental stages (Dykens et al., 2004; Levitt et al., 2004; Polleux and Lauder, 2004; Rubenstein and Merzenich, 2003). In this respect, a cluster of the GABA_A_ receptor subunits in the duplicated region and its relevance to development is of particular interest for further study. It remains possible that other genes in this duplicated region and their downstream effects may cause abnormal behavior. Systematic approaches such as using a series of BACs tiled across the region to make transgenic mice will help to resolve these questions. Our model mouse will be valuable not only for therapeutic studies but also provides a starting point for more detailed genetic analysis directed toward understanding the etiology of developmental brain disorders.

## Experimental Procedures

### A Chromosome-Engineered Mouse Model

The detailed procedure of the Cre/*loxP* chromosomal engineering system was described previously (van der Weyden and Bradley, 2006; Zheng et al., 1999). Genomic DNA was derived from male 129S5 mice. The 5′*hprt* (hypoxanthine phosphoribosyl transferase) library vector carries the neomycin resistance gene for gene targeting, a *loxP* site, the 5′*hprt* minigene for chromosome engineering, and a Tyrosinase minigene for coat color tagging. The 3′*hprt* library backbone contains the puromycin resistance gene, a *loxP* site, 3′*hprt*, and an Agouti transgene under the control of the K14 promotor. Each rearrangement requires the successive targeting of two end points with complementary halves of the *Hprt* minigene and different positive selection marker. By recombination, the *Hprt* minigene is reconstituted so that cell with rearranged chromosomes can be selected with HAT media. The 5′*hprt* and 3′*hprt* libraries were screened with a 440 bp fragment between mouse *Mkrn3* and *Frat3* genes and a 930 bp fragment between *Herc2* and *Shyc* genes, respectively. Two targeting vectors were sequentially transfected into AB2.2 *hprt*-deficient embryonic stem (ES) cells by electroporation, confirming the structure of the recombinant chromosome at each step by Southern blotting after drug selection with G418 or puromycin. The double-targeted ES cells were used to induce the rearrangement. The Cre expression vector pOG231 was electroporated into these cells, and the recombination products were selected with HAT medium. The clones carrying the duplication were injected into 3.5 day blastocysts from C57BL/6-*Tyr^cBrd^/^cBrd^* mice. Chimaeras that are generated from blastocyst injection are mated with C57BL/6- *Tyr^cBrd^/^cBrd^* WT mice to establish germline transmission of the modified genome. The experimental procedures and housing conditions for animals were approved by the Osaka Bioscience Institute Animal Research Committee.

### CGH by BAC Microarray

The mouse whole-genome BAC array used in this study contained 2803 unique BAC clones from mouse genomic libraries spaced at 1 Mb intervals (Chung et al., 2004). Detailed conditions are included in the Supplemental Experimental Procedures.

### Quantitative Real-Time Reverse Transcription PCR

The quantitative assays for mRNA expression were described previously (Yamamoto et al., 2005). TaqMan Low Density Array (Applied Biosystems), which contained mouse *Tubgcp5*, *Herc2*, *P*, *Gabrγ3*, *Gabrα5*, *Gabrβ3*, *Atp10a*, *Ube3a*, *Magel2*, *Mkrn3*, *Chrna7*, and 18S rRNA (internal control), was examined with an ABI PRISM 7900HT Sequence Detection System (Applied Biosystems). Each quantification of relative RNA levels by the SYBR Green real-time PCR technology was done as described previously (Akashi and Takumi, 2005). The PCR primers and detailed procedures are included in the Supplemental Experimental Procedures.

### Three-Chambered Social Interaction

Social testing apparatus consisted of a rectangular, three-chambered box and a lid with an infrared video camera (Nadler et al., 2004) (Ohara & Co., Tokyo). Each chamber was 20 × 40 × 22 cm, and the dividing walls were made from clear Plexiglas, with small square openings (5 × 3 cm) allowing access into each chamber. An unfamiliar C57BL/6J male (stranger) that had no prior contact with subject mice was placed in one of the side chambers. The location of stranger in the left versus right side chamber was systematically alternated between trials. The stranger mouse was enclosed in a small, round wire cage, which allowed olfactory, visual, auditory, and tactile contacts but did not allow sexual and deep contacts. The subject mouse was first placed in the middle chamber and allowed to explore the entire social test box for a 10 min session. Measures were taken of the amount of time spent in quadrant around wire cage by a camera, which is attached at the top of box. More detailed conditions are included in the Supplemental Experimental Procedures.

### Morris Water Task

The visible platform, hidden platform, probe test, and reversal probe test components of the Morris water task were conducted in a circular pool, 1.0 m in diamater (Ohara & Co.). Detailed conditions are included in the Supplemental Experimental Procedures.

### Barnes Maze Task

The Barnes maze task was conducted on “dry land,” a white circular surface, 1.0 m in diameter, with 12 holes equally spaced around the perimeter (Miyakawa et al., 2001) (Ohara & Co.). Detailed conditions are included in the Supplemental Experimental Procedures.

### Ultrasonic Vocalization

After habituation, each pup was removed from its mother and placed in a stainless steel cylinder (size 7.5 cm diameter × 7 cm height) on the COOL PLATE (NCP-2215, Nisshin Rika Co., Ltd.) which maintained temperature of the cylinder at 24°C in a sound proof room (AT-81, RION Co., Ltd.). The number of vocalizations was measured for 5 min. More detailed conditions are included in the Supplemental Experimental Procedures.

### Calcium Measurement in Neuronal Cell Culture

The procedure for primary culture of neurons was described previously (Yoshimura et al., 2006). The neurons were prepared from embryonic mice brain at E16 and plated onto poly-L-lysine-coated glass bottom dishes. All measurements were performed within 7–9 days from preparation. For measurement of the intracellular calcium, primary cultured neurons were loaded with 5 μM Fura-2 acetoxymethyl ester (Dojindo) at room temperature for 30 min. Cells on a coverglass placed in a recording chamber were perfused with HEPES solution (135 mM NaCl, 5 mM KCl, 2 mM CaCl_2_, 2 mM MgCl_2_, 10 mM HEPES, and 10 mM glucose, adjusted at pH 7.4 with NaOH) by gravity. This chamber was mounted on the stage of an inverted fluorescence microscope (Axiovert 135, Zeiss). Various concentrations (0.1 nM, 1 nM, 10 nM, 100 nM, and 1000 nM) of WAY 161503 (Rosenzweig-Lipson et al., 2006) (Tocris) were perfused for 3 min with a 10 min interval. With a digital image analysis system (MetaFluor, Molecular Devices), the fluorescence ratio (340 nm/380 nm) for each neuron was analyzed. For data analysis, the neurons displaying above 0.02 on the intensity (Δ 340 nm/380 nm) at 1 μM WAY 161503 were selected.

### Statistical Analysis

Statistical analysis was conducted with StatView (SAS institute). Data were analyzed by two-way ANOVA, or two-way repeated-measures ANOVA, or one-way ANOVA followed by Bonferroni-Dunn test unless noted otherwise. Values in tables and graphs were expressed as mean ±SEM.

### Other Methods

All the detailed procedures were included in the Supplemental Experimental Procedures.

## Supplemental Data

Document S1. Supplemental Experimental Procedures, 15 Figures, and Two Tables
