# Supplementary material for: Abnormal Behavior in a Chromosome- Engineered Mouse Model for Human 15q11-13 Duplication Seen in Autism
Source: Cell. 2009 Jun 26;137(7):1235–46. doi: 10.1016/j.cell.2009.04.024 (PMC3710970; doi:10.1016/j.cell.2009.04.024)
Supplement: Document S1. Supplemental Experimental Procedures, 15 Figures, and Two Tables [file mmc1.pdf]

Cell, Volume *137*

Supplemental Data

Abnormal Behavior in a Chromosome-Engineered Mouse Model for Human 15q11-13 Duplication Seen in Autism

Jin Nakatani, Kota Tamada, Fumiyuki Hatanaka, Satoko Ise, Hisashi Ohta, Kiyoshi Inoue, Shozo Tomonaga, Yasuhito Watanabe, Yeun Jun Chung, Ruby Banerjee, Kazuya Iwamoto, Tadafumi Kato, Makoto Okazawa, Kenta Yamauchi, Koichi Tanda, Keizo Takao, Tsuyoshi Miyakawa, Allan Bradley, and Toru Takumi

Supplemental Experimental Procedures

Southern blot. Genomic DNAs were isolated from mouse ES cells by the proteinase K/SDS digestion. DNA was electrophoresed on a 0.8% agarose gel and transferred to a Hybond-N+ membrane (Amersham). The membrane was hybridized with α^{32}P-dCTP labeled probes at 65 ^{o}C. The DNA probes used were a 330-bp fragment between mouse *Herc2* and *Shyc* genes (probe A) and a 700-bp fragment between *Mkrn3* and *Peg12* (probe B). The filter was washed in 0.6x SSC and 1% SDS at 65 ^{o}C.

Animals. The original experiments except the following ones were conducted by using 129SvEv background mice (Figure 2, 3, S1-4, S6-8, S11-12, S14-15). Mice backcrossed with C57BL/6J were used in 3-chamber test, Morris water maze task, Barnes maze test, all USV tests, T-maze test, olfactory immunohistochemistry, and olfactory habituation/dishabituation test (Figure 4, 5, 6, S5, S9-10, S13).

Fluorescence *in situ* hybridization (FISH). Chromosome spreads of ES cells for hybridization were prepared as described (Robertson, 1987). RP23-325M1 was labeled with biotin and detected avidin-Texas Red. RP23-270C2 was labeled with digoxigenin and detected by anti-digoxigenin-FITC.

CGH by BAC microarray. Genomic DNA was extracted and purified by standard protocol. Test DNA and control DNA were digested with *Hae*III for 2 h. Digested genomic DNA was labeled with Cy3- or Cy5-dCTP by random priming (BioPrime DNA Labeling Kit, Invitrogen). Hybridization was performed in humid chamber on an orbital shaker for 48 h at 37 ^{o}C. Slides were washed in PBS containing 0.05% Tween 20 for 10 min at room temperature, next in 50% formamide 2x SSC at 42 ^{o}C for 30 min, and last in same condition as first wash. Finally, slides were spin-dried for 3 min at 1000 rpm.

Quantitative real-time reverse transcription (RT)-PCR. For one port of the TaqMan Low Density Array, 100 ng cDNA template was mixed with 50 µl of 2x TaqMan Universal PCR Master Mix (Applied Biosystems) and filled up to 100 µl with distilled water. The reaction was first incubated at 50 ^{o}C for 2 min, then at 95 ^{o}C for 10 min, followed by 40 cycles of 95 ^{o}C for 15 sec and 60 ^{o}C for 1 min. The PCR primers for the SYBR Green real-time PCR were designed with Primer express software (Applied Biosystems), and sequences of primers were as follows: *Ndn*

FW: 5'-GTA TCC CAA ATC CAC AGT GC-3', *Ndn* RV: 5'-TAA CTC TCC AGG GCC TTC TT-3', *Snrpn* FW: 5'-GCA AAA CAG CCA GAA CGT GAA-3', *Snrpn* RV: 5'-GCA CAC GAG CAA TGC CAG TAT-3', 18S rRNA FW: 5'-CGC CGC TAG AGG TGA AAT TC-3', 18S rRNA RV: 5'-CGA ACC TCC GAC TTT CGT TCT-3', 18S rRNA TaqMan probe: CCG GCG CAA GAC GGA CCA GA. For a 25-µl PCR reaction, 50 ng cDNA template was mixed with the primers and probe to final concentrations of 300 nM and 2 nM, respectively, and 12.5 µl of 2x TaqMan PCR Master Mix (Applied Biosystems). The reaction was first incubated at 50 °C for 2 min, then at 95 °C for 10 min, followed by 40 cycles of 95 °C for 15 sec and 60 °C for 1 min.

***in situ* hybridization.** The procedure was performed as following our previous report (Inoue et al., 2004). The 10-week-old C57BL/6J mice were sacrificed by cervical dislocation, and their brains were removed, immediately frozen in powdered dry ice, and stored at –70 °C. Twenty µm cryosections were cut on a cryostat at –15 °C and mounted onto APS-coated slides (Matsunami, Osaka). Slides were stored at –20 °C until use. Template DNAs for probes were synthesized by PCR using specific primers and mouse brain cDNA. Digoxigenin (DIG)-labeled RNA probes were synthesized by *in vitro* transcription using sequence for *Snrpn* (the sequence corresponding to the nucleotides 449-1187 of Gene accession number NM-013670; for *Ube3a*, nucleotides 678-1371 of NM-011668; for *Gabrα5*, nucleotides 343-1250 of NM-176942) as templates. In addition, sense probe for the templates was synthesized as negative control. Sections were fixed with 4% paraformaldehyde in phosphate buffered saline (PBS) pH 7.4, treated with proteinase K (5 µg/ml) for 3 min, fixed with 4% paraformaldehyde/0.2% glutaraldehyde in PBS for 15 min, and prehybridized at 70 °C for 1 h with hybridization buffer (50% formamide, 5x SSC, 50 µg/ml yeast tRNA, 1% SDS, and 50 µg/ml heparin). The sections were then incubated for 12 h at 70 °C in the same hybridization buffer containing the DIG-labeled probe. The sections were washed with 50% formamide, 5x SSC, 1% SDS at 70 °C for 45 min, 50% formamide, 2x SSC at 65 °C for 45 min, and Tris-buffered saline containing 0.1% Tween-20 (TBST) for 30 min. The sections were subsequently incubated in 0.5% blocking reagent (Roche Diagnostics, Basel, Switzerland) in TBST for 1 h, and in the same solution containing an alkaline phosphatase-conjugated anti-DIG antibody (Roche Diagnostics) at 1:2000 for 4 h. Sections were next washed in TBST, and then in 100 mM NaCl, 100 mM Tris-HCl, pH 9.5, 50 mM $MgCl_2$, 0.1% Tween-20 (NTMT). Sections were then incubated with color reagents, 175 µg/ml 5-Bromo-4-chloro-3-indolyl-phosphate, 450 µg/ml 4-Nitro blue-tetrazolium chloride, and 1 mM Levamisole in NTMT for 6 h. Slides were washed with PBS containing 0.1% Tween-20, dehydrated in graded ethanols, cleared three times in xylene, and cover-slipped with Entellan. Photographs of brain sections were captured with a digital camera DP70 (Olympus, Tokyo, Japan).

Histology. Adult animals were anesthetized with Nembutal (pentobarbital sodium, 0.2 mL/100 g body weight), and fixed by transcardial perfusion with 4% paraformaldehyde in 0.1 M phosphate buffer (PB, pH 7.4). Brains were removed from the skull, embedded in paraffin. Paraffin-embedded brains were coronally sectioned at 4- or 8-µm thickness on a microtome, and mounted on APS-coated slides. For 4-µm thickness sections, every fifth sections were deparaffinized, and stained with Hematoxylin and Eosin (Merck). Stained sections were dehydrated in an ethanol series, cleaned in xylene, and then coverslipped. For 8-µm thickness, every fifth sections were also deparaffinized and stained with Bodian staining. In brief, the sections were immersed in 1% potassium dichromate for 1 h at room temperature, and then soaked in 1% silver protein (Merck) for 24 h at 37 °C. The sections were washed in the reduction

solution (1 g hydroquinone, 5.6 g sodium sulphate, 140 ml distilled water) for 10 min, and then toned in 1% gold chloride solution for 1 h. After washing, the sections were immersed in 2% oxalic acid solution for 5 min, and then soaked in 5% sodium thiosulphate solution. After rinse, the sections were dehydrated, cleared in xylene and mounted. Images were obtained with an upright light microscope equipped with a digital camera (Olympus, DP70).

Counting the number of Purkinje cells. Five micrometer coronal sections stained with Hematoxylin and Eosin were used for the quantification. The number of Purkinje cells in 6 Simple lobules was counted along 500 μm of Purkinje-cellular layer. Only the cells with a cell body in focus were counted manually. For blind analysis, name of the files were substituted with a random number.

Immunohistochemistry. Mice were anesthetized with 0.1 mg/g pentobarbital sodium and transcardially perfused with ice-cold PBS, followed by ice-cold 4% paraformaldehyde in phosphate buffer for 15 min. Olfactory mucosa and brain were dissected out and postfixed in 4% paraformaldehyde for 2 h at 4°C. Olfactory mucosa was subsequently decalcified with Morse's solution (10% w/v sodium citrate and 22.5 % v/v formic acid) for 2 days with moderate stirring at 4°C. Brain and olfactory mucosa were cryoprotected in 30% sucrose overnight at 4°C, embedded in OCT (Tissue Tek) and frozen on powdered dry ice. Serial coronal sections at 16 μm for olfactory mucosa and at 30 μm for olfactory bulbs were made with a cryostat and collected on MAS coated slides (Matsunami) and stored at -80°C until use. Immunohistochemistry was performed as follows: sections were washed with PBS for 5 min, treated with 0.3% H_2O_2 for 30 min, rinsed in PBS for 5 min for 3 times, incubated with a blocking solution (PBS containing 5% normal horse serum and 0.3% Triton X-100) for 30 min, incubated with primary antibodies diluted in the blocking solution overnight at 4°C, washed with PBS containing 0.3% Triton X-100 (PBST) for 10 min for 3 times, incubated with biotinylated secondary antibodies diluted in blocking solution for 1 h to form immune complexes, and washed with PBST for 10 min and PBS for 10 min for 2 times. Immune complex signals were visualized using the Vectastain ABC standard kit (Vector). The primary antibodies and their dilution ratio used in this study were anti-OMP antibody, 1:2000 (Wako Chemical) and anti-Reelin antibody, 1:1000 (Calbiochem). For olfactory bulbs, antigen retrieval process was carried out by heating the sections for 40 min at 95°C in 10 mM sodium citrate (pH 6.0), kept at room temperature for 20 min and washed two times with PBS.

Methylation analysis. Genomic DNAs isolated from mouse tails were digested with either *Hpa*I alone or in combination with *Bss*HII. Southern blot analysis of differential methylation at the *Snurf-Snrpn* loci were hybridized with a 1.3-kb fragment from *Snurf* intron 1.

General health and neurological screen. A general health and neurological screen was conducted as previously described (Miyakawa *et al.*, 2001). The righting, whiskers touch, and ear twitch reflexes were evaluated, and a number of physical features including body weight and temperature, and the presence of whiskers or bald hair patches were recorded.

Neuromuscular examination. The neuromuscular strength was examined by the grip strength and wire-hanging tests. The grip strength meter (O'Hara & Co.) was used to assess forelimb grip strength. Mice were lifted and held by their tail so that their forepaws could grasp a wire grid.

Mice were then gently pulled backward by the tail with their posture parallel to the surface of the table until they release the grid. The peak force applied by mouse forelimbs was recorded in Newton (Takao and Miyakawa, 2006). Each mouse was tested three times and the greatest value measured was used for statistical analysis. In the wire hang test, mice were placed on a wire mesh, which was then inverted and waved gently, so that the subject gripped the wire. Latency to fall was recorded, with a 60 sec cut-off time.

Light/dark transition test. The apparatus used for light/dark transition test consisted of a cage (21×42×25 cm) divided into two sections of equal size by a partition with door (O'Hara & Co., Tokyo). One chamber was brightly illuminated (390 lx), whereas the other chamber was dark (2 lx). Mice were placed into the dark side, and allowed to move freely between the two chambers with door open for 10 min. The total number of transitions, time spent in each side, first latency to light side, and distance traveled were recorded automatically using Image LD software (see 'Image analysis'). On-line material describing this method is available visually (Takao and Miyakawa, 2006).

Open field test. Locomotor activity was measured using an open field test. Each subject was placed in the center of the open field apparatus (40×40×30 cm; Accuscan Instruments, Columbus, Ohio). Total distance traveled (centimeters), vertical activity (rearing measured by counting the number of photobeam interruptions), time spent in the center, the beam-break counts for stereotyped behaviors and number of fecal boli were recorded. Data were collected for 120 min.

Hot plate test. The hot plate test was used to evaluate the nociception or the sensitivity to a painful stimulus. Mice were placed on a 55.0±0.3 ˚C hot plate (Columbus Instruments, Columbus, Ohio), and latency to the first hind-paw response was recorded. The hind-paw response was either a foot shake or a paw lick.

Social interaction test in a novel environment (one-chamber). Two mice of identical genotypes, which were previously housed in different cages, were placed into a box together (40×40×30 cm) and allowed to explore freely for 10 min. Social behavior was monitored by a CCD camera, which was connected to a Macintosh computer. Analysis was performed automatically using Image SI software. The number of contacts, the duration of contacts, and total distance traveled were measured.

Rotarod test. Motor coordination and balance were tested with the rotarod test. The rotarod test using a rotarod (UGO Basile Accelerating Rotarod) was performed by placing a mouse on a rotating drum (3 cm diameter) and measuring the time each animal was able to maintain its balance on the rod. The mouse was given two practice trials and the place on the rotating cylinder. During the trial, each mouse was placed on the accelerated rotarod for a maximum of 300 sec, and the mean latency to fall off the rotarod (for the five trials) was recorded and used in subsequent analysis.

Startle response/prepulse inhibition tests. A startle reflex measurement system was used (O'Hara & Co., Tokyo). A test session began by placing a mouse in a plexiglas cylinder where it was left undisturbed for 10 min. The duration of white noise that was used as the startle stimulus was 40 msec for all trial types. The startle response was recorded for 140 msec (measuring the

response every 1 msec) starting with the onset of the prepulse stimulus. The background noise level in each chamber was 70 dB. The peak startle amplitude recorded during the 140 msec sampling window was used as the dependent variable. A test session consisted of 6 trial types (i.e. two types for startle stimulus only trials, and four types for prepulse inhibition trials). The intensity of startle stimulus was 110 or 120 dB. The prepulse sound was presented 100 msec before the startle stimulus, and its intensity was 74 or 78 dB. Four combinations of prepulse and startle stimuli were employed (74-110, 78-110, 74-120, and 78-120). Six blocks of the 6 trial types were presented in pseudorandom order such that each trial type was presented once within a block. The average inter-trial interval was 15 sec (range: 10-20 sec).

Eight-arm radial maze test. Fully-automated eight-arm radial maze apparatuses (O'Hara & Co., Tokyo, Japan) were used. The floor of the maze was made of white plastic, and the wall (25 cm high) consisted of transparent plastic. Each arm (9 x 40 cm) radiated from an octagonal central starting platform (perimeter 12 x 8 cm) like the spokes of a wheel. Identical food wells (1.4 cm deep and 1.4 cm in diameter) with pellet sensors were placed at the distal end of each arm. The pellets sensors were able to automatically record pellet intake by the mice. The maze was elevated 75 cm above the floor and placed in a dimly-lit room with several extra-maze cues. During the experiment, the maze was maintained in a constant orientation. One week before pretraining, animals were deprived of food until their body weight was reduced to 80% to 85% of the initial level. Pretraining started on the 8th day. Each mouse was placed in the central starting platform and allowed to explore and consume food pellets scattered on the whole maze for a 30-min period (one session per mouse). After completion of the initial pretraining, mice received another pretraining to take a food pellet from each food well after being placed at the distal end of each arm. A trial was finished after the mouse consumed the pellet. This was repeated eight times, using eight different arms, for each mouse. After these pretraining trials, actual maze acquisition trials were performed. In the spatial working memory task of the eight-arm radial maze, all eight arms were baited with food pellets. Mice were placed on the central platform and allowed to obtain all eight pellets within 25 min. A trial was terminated immediately after all eight pellets were consumed or 25 min had elapsed. An 'arm visit' was defined as traveling more than 5 cm from the central platform. The mice were confined at the center platform for 5 s after each arm choice. The animals went through one trial per day. For each trial, arm choice, latency to obtain all pellets, distance traveled, number of different arms chosen within the first eight choices, the number of arm revisited, and omission errors were automatically recorded. In the reference memory task of the eight-arm radial maze, one of the eight arms was consistently baited with one food pellet in the food well and a trial was terminated immediately after the one pellet was consumed. Data acquisition, control of guillotine doors, and data analysis were performed by Image RM software (see 'Image analysis').

Tail suspension test. The tail suspension test was performed for a 10-min test session according to toe procedures described previously (Steru et al., 1985). Mice were suspended 30 cm above the floor in a visually isolated area by adhesive tape placed approximately 1 cm from the tip of the tail, and their behavior was recorded over a 10-min test period. Data acquisition and analysis were performed automatically, using Image TS software.

Twenty-four hour home cage monitoring. Social interaction monitoring in home cage was

conducted as previously described (Miyakawa et al., 2003). To monitor social behavior between two mice in a familiar environment, a system that automatically analyzes social behavior in home cages of mice was developed. The system contains a home cage (29 x 18 x 12 cm) and a filtered cage top, separated by a 13-cm-high metal stand containing an infrared video camera, which is attached at the top of the stand. Two mice of the same inbred strain that had been housed separately were placed together in a home cage. Their social behavior was then monitored for a week. Outputs from the video cameras were fed into a Macintosh computer. Images from each cage were captured at a rate of one frame per second. Social interaction was measured by counting the number of particles in each frame: two particles indicated the mice were not in contact with each other; and one particle demonstrated contact between the two mice. We also measured locomotor activity during these experiments by quantifying the number of pixels changed between each pair of successive frames. Analysis was performed automatically using ImageHA software (see 'Image analysis').

Contextual and cued fear conditioning. Each mouse was placed in a test chamber (26 x 34 x 29 cm) inside a sound-attenuated chamber and allowed to explore freely for 2 min. A 60 dB white noise, which served as the conditioned stimulus (CS), was presented for 30 sec, followed by a mild (2 sec, 0.5 mA) foot shock, which served as the unconditioned stimulus (US). Two more CS-US pairings were presented with 2 min inter-stimulus interval. Context testing was conducted 24 h after conditioning in the same chamber. Cued testing with altered context was conducted after conditioning using a triangular box (35 x 35 x 40 cm) made of white opaque plexiglas, which was located in a different room. Data acquisition, control of stimuli (i.e. tones and shocks), and data analysis were performed automatically, using Image FZ software. Images were captured at 1 frame per second. For each pair of successive frames, the amount of area (pixels) by which the mouse moved was measured. When this area was below a certain threshold (i.e. 20 pixels), the behavior was judged as ‘freezing’. When the amount of area equaled or exceeded the threshold, the behavior was considered as ‘non-freezing’. The optimal threshold (amount of pixels) to judge freezing was determined by adjusting it to the amount of freezing measured by human observation. ‘Freezing’ that lasted less than the defined time threshold (i.e. 2 sec) was not included in the analysis.

Elevated plus maze test. The elevated plus-maze consisted of two open arms (25 x 5 cm) and two enclosed arms of the same size, with 15 cm high transparent walls. The arms and central square were made of white plastic plates and were elevated to a height of 55 cm above the floor. In order to minimize the likelihood of animals falling from the apparatus, 3-mm high plexiglas ledges were provided for the open arms. Arms of the same type were arranged at opposite sides to each other. Each mouse was placed in the central square of the maze (5 x 5 cm), facing one of the closed arms. Mouse behavior was recorded during a 10-min test period. Time spent on open arms was recorded. Data acquisition and analysis were performed automatically, using Image EP software.

Porsolt forced swim test. The apparatus consisted of four plexiglas cylinders (20 cm height x 10 cm diameter). The cylinders were separated from each other by a nontransparent panel to prevent mice from seeing each other. The cylinders were filled with water (23 ^{o}C), up to a height of 7.5 cm. Mice were placed into the cylinders, and their behavior was recorded over a 10-min test period. Data acquisition and analysis were performed automatically, using Image PS software (see Image

Analysis). Immobility was measured by Image OF software (see Image Analysis) using stored image files.

Three-chambered social interaction (2). As for a novel inanimate object vs. empty, a dodecahedral pole with black and white rectangle faces alternately was used for an inanimate object and was placed in a wire cage in one side chamber. As for another inanimate object vs. a novel mouse, a black cone was used for an inanimate object and an adult conspecific mouse (C57BL/6J) that has had no previous contact with the subject (test mouse) was used for a novel mouse. As for a novel vs. familiar mouse, another adult conspecific mouse (C57BL/6J) that has had no previous contact with the subject (test mouse) was used for a novel mouse and the same mouse that was examined in the previous test was used for a familiar mouse.

Morris water task. The water maintained at room temperature (20-23 degrees) was rendered opaque by the addition of nontoxic white paint. Videotracking was conducted with a camera focused on the full diameter of the pool. Each training trial began by placing the mouse into the quadrant that was either right of, left of, or opposite to the target quadrant containing the platform, in semirandom order. Different order of the start positions was applied every day, but the identical order of start positions was used for all subjects. Training trials were 60 s maximum duration. A mouse that failed to reach the platform within 60 s was subsequently guided to the platform. Latency to reach the platform, distance traveled to the platform, average swim speed, and percent time spent at the perimeter of the pool (thigmotaxis) were automatically recorded. Four trials per day were conducted for 4 successive days for acquisition of the visible platform task. Four trials per day were conducted for 7 successive days for acquisition of the hidden platform task with the original platform location and 4 successive days with a new platform location (reversal probe test) rotated by 180-degree from the original platform location. At the end of the seventh and eleventh days of hidden platform training, a probe test and a reversal probe test was conducted for 1 min to confirm that this spatial task was acquired based on navigation by distal environmental room cues, respectively. Time spent in each quadrant, number of crossings above the former target site, average speed, and percent time spent at the perimeter of the pool were automatically recorded.

Barnes maze task. The circular open field was elevated 75 cm from the floor. A black Plexiglas escape box (17×13×7 cm), which had paper cage bedding on its bottom, was located under one of the holes. The hole above the escape box represented the target, analogous to the hidden platform in the Morris task. The location of the target was consistent for a given mouse but randomized across mice. The maze was rotated daily, with the spatial location of the target unchanged with respect to the distal visual room cues, to prevent a bias based on olfactory or the proximal cues within the maze. Three trials per day were conducted for 6 successive days. On day 7, a probe trial was conducted without the escape box, to confirm that this spatial task was acquired based on navigation by distal environment room cues. As for reversal task, the location of target for each mouse was shifted to the complete opposite side on circular surface. Same trials were also carried out and conducted a probe trial. Time spent around each hole was recorded by videotracking software (Image BM).

T-maze test. Left-right discrimination task was conducted using an automatic T-maze (Tujimura et al., 2008). It was constructed of white plastics runways with walls 25-cm high. The maze was partitioned off into 6 areas by sliding doors that can be opened downward. The stem of T was

composed of area S2 (13×24 cm) and the arms of T were composed of area A1 and A2 (11.5×20.5 cm). Area P1 and P2 were the connecting passage way from the arm (area A1 or A2) to the start compartment (area S1). The end of each arm was equipped with a pellet dispenser that could provide food reward. The pellet sensors were able to record automatically pellet intake by the mice. One week before the pre-training, mice were deprived of food until their body weight was reduced to 80-85% of the initial level. Mice were kept on a maintenance diet throughout the course of all the T-maze experiments. Before the first trial, mice were subjected to three 10-min adaptation sessions, during which they were allowed to freely explore the T-maze with all doors open and both arms baited with food. On the day after the adaptation session, mice were subjected to a left-right discrimination task for 6 days (one session consisting of 10 trials, 2 sessions per day; cutoff time, 50 min). Mice were given 10 pairs of training trials per day. The mouse was able to freely choose either the right or left arm of the T (area A1 or A2). Correct arm was assigned to each mouse randomly. If a mouse chose the correct arm, mouse could receive a reward at the end of the arm. Choosing an incorrect arm resulted in no reward and confinement to the arm for 10 sec. After the mouse consumed the pellet or the mouse stayed more than 10 sec without consuming the pellet, door that separated the arm (area A1 or A2) and connecting passage way (area P1 or P2) would be opened and the mouse could return to the starting compartment (area S1), via connecting passage way. On the 7th day, the correct arm was changed to the opposite for reversal learning. A variety of fixed extra-maze clues surrounded the apparatus.

Image analysis. All applications used for the behavioral studies (Image EP, Image BM, Image SI and Image PS) were run on a Macintosh computer. Applications were based on the public domain NIH Image or Image J program (developed by Wayne Rasband at the U.S. National Institute of Mental Health and available on the Internet at http://rsb.info.nih.gov/nih-image/) and were modified for each test by Tsuyoshi Miyakawa (available through O'Hara & Co.).

Ultrasonic vocalization. Both male and female pups (P5–22) were used. Pups were raised with their mother and littermates in a cage placed in an animal holding room (room temperature: 23.0 ± 2.0 ºC, humidity: 55.0 ± 15.0%) with a 12 h light-dark cycle (lights on at 07:00). On the day of the study, the pups with their mother were moved to the experimental room at least 60 min before the initiation of the study. After habituation, each pup was removed from its mother and placed in a stainless-steel cylinder (size 7.5 cm diameter x 7 cm height) on the COOL PLATE® (NCP-2215, Nisshin Rika Co., Ltd.) which maintained temperature of the cylinder at 24 ºC in a sound proof room (AT-81, RION Co., Ltd.). The number of vocalization was measured for 5 min. Individual calls made by each pup were collected by an ultrasound detectable microphone (UC-29, RION Co., Ltd.), and then amplified by a preamplifier (NH-05A, RION Co., Ltd.) and a main amplifier (UN-04A, RION Co., Ltd.) with a filter (Multifunction filter 3611, NF Corporation Co., Ltd., settings: high pass 1.5 kHz). Then analog signals were converted to digital signals by an A/D converter (CH-3150, Exacq Technologies, Inc.), and stored in a personal computer (PRECISION 470®, Dell Inc.). The numbers of vocalizations were counted by the recording software (Dasy Lab® 9.0, measX GmbH and Co. KG.) with a digital filter at 20 kHz to count the vocalizations generated at ultrasound range. The threshold value was set at a signal amplitude of 0.05 V to exclude noise. Measurements of vocalizations were conducted at 5, 7, 14 days and 3 weeks (21 or 22 days) after the birth. Each pup was immediately returned to its mother in the home cage after the measurement. Surface body temperature of each pup was monitored with an infrared thermometer (Thermo-Hunter PT3S, Optex Co., Ltd.) immediately before and after the isolation.

In the case of power spectrum analysis, stored signals were transformed with the fast Fourier transformation method by the software.

Vocalization in a resident-intruder paradigm. Male mice (14 WT and 14 *patDp/+* mice, 7-8 weeks) were used. Resident mice were individually housed for 4 weeks before the test session. Intruder mice were maintained in social groups of three to four per home cage for 1 week before the test session. Body weights of resident and intruder mice were comparable (WT: resident vs. intruder; 20.6 +/- 0.5 g vs. 21.4 +/- 0.4 g, *patDp/+*: resident vs. intruder; 22.8 +/- 0.5 g vs. 21.8 +/- 1.0 g). On the day of the study, the animals were moved to the experimental room from the animal room and left for at least 60 min before initiation of the study. After habituation, the cage with a resident mouse was placed under an ultrasound detectable microphone in a sound proof room. Both audible (frequency between 1.5 kHz and 20 kHz) and ultrasonic (above 20 kHz) vocalizations were recorded with the same system for maternal separation study except using both 1.5kHz and 20 kHz filters to separate ultrasonic calls from total calls, because an adult mouse emitted calls ranging from audible to ultrasonic bands. A pair of resident and intruder mice was made from the same genotyped mice. An intruder was introduced into the home cage of a resident mouse for 10 min. Total calls emitted by a pair of resident and intruder mice for the last 5 min were recorded. Resident showed agonistic behavior against intruder in the first 5 min, thus vocalization during this period may be affected by the high level of emotion. After measurement, an intruder mouse was removed from the home cage of a resident mouse. The test was conducted between 13:00 and 17:00.

Olfactory habituation/dishabituation test. The ability of the mice to detect social and non-social odors was examined with the olfactory habituation/dishabituation method as previously described (Crawley et al., 2007) with some modifications. The animal was transferred to the testing room and reared individually 6 h prior to testing. One hour before the test, for acclimatizing the swab itself, swab was adhered with the tape on each cage lid where mice could not contact directly because of the existence of wire bars of lid between them. The swab tip was placed at a level 5 cm from the bottom of the cage. All testings were done in the light, but during the dark phase of the light cycle. During the test, food and water were deprived and lid was changed to a clean one. Odorant stimuli were ultrapure water, cage of female mice, banana extract (Golden Kelly Patent Flavor Co, Ltd., Osaka, Japan) diluted 1:10 in ultrapure water, and almond extract (Golden Kelly Patent Flavor Co, Ltd.) diluted 1:10 in ultrapure water. The choice of these odorants and dilutions was based on data from pilot experiments in our laboratory. Experimenters prepared stimuli by dipping a swab into the stimulus solution (water, banana, and almond) or by wiping in a zig-zag pattern across the floor of the dirty cage of female mice by the water-soaked swab. The swab was consequently adhered by the tape on the cage lid at a level 5 cm from the bottom of the cage. Each stimulus was presented for 2 min and then replaced by a new applicator, three times in succession for a total of 12 presentations. The order of presentation was water, cage, banana and almond. The behavior was monitored by an 8-mm video camera and was recorded on a PC. By using the recorded data, the amount of time the subject spent with its nose above the wire bars beneath the swab was counted by stopwatch later.

RNA blot hybridization. RNAs were isolated from mouse brains by SV total RNA isolation system (Promega). One μg of total RNA was electrophoresed on a 1% formaldehyde gel and transferred to a Hybond-N+ membrane (Amersham). The membrane was hybridized with

γ^{32}P-ATP labeled MBII52 probe (72-bp) and α^{32}P-dCTP labeled G3PDH probe at 65 ^{o}C. The filter was washed in 0.6 x SSC and 1% SDS at 65 ^{o}C. BAS-2000 (Fuji Photo Film) was used for imaging and its quantification.

Pyrosequencing. The pyrosequencing analysis was conducted according to the previous report (Iwamoto et al., 2005). Four µl of streptavidin-sepharose beads (Amersham) and 29 µl of binding buffer (10 mM Tris-HCl, 1 mM EDTA, 2 M NaCl, 0.1% Tween 20 at pH 7.6) were mixed with 25 µl of RT-PCR product for 10 min at room temperature. The reaction mixture was placed onto a MultiScreen-HV, Clear Plate (Millipore). After applying the vacuum, the beads were treated with a denaturation solution (0.2 N NaOH) for 1 min, and washed twice with washing buffer (10 mM Tris-Acetate at pH 7.6). The beads were then suspended in 50 µl of annealing buffer (20 mM Tris-Acetate, 2 mM Mg-Acetate at pH 7.6) containing 10 pmol of sequencing primer. The template-sequencing primer mixture was transferred onto a PSQ 96 Plate (Biotage), heated to 90 ^{o}C for 2 min, and cooled to room temperature. Sequencing reactions were performed with a PSQ 96 SNP Reagent Kit (Biotage) using a PSQ96MA (Biotage) according to the manufacture's instructions.

Supplemental References

Crawley, J.N., Chen, T., Puri, A., Washburn, R., Sullivan, T.L., Hill, J.M., Young, N.B., Nadler, J.J., Moy, S.S., Young, L.J., *et al.* (2007). Social approach behaviors in oxytocin knockout mice: comparison of two independent lines tested in different laboratory environments. Neuropeptides *41*, 145-163.

Inoue, K., Terashima, T., Nishikawa, T., and Takumi, T. (2004). Fez1 is layer-specifically expressed in the adult mouse neocortex. Eur J Neurosci *20*, 2909-2916.

Iwamoto, K., Bundo, M., and Kato, T. (2005). Estimating RNA editing efficiency of five editing sites in the serotonin 2C receptor by pyrosequencing. Rna *11*, 1596-1603.

Miyakawa, T., Leiter, L.M., Gerber, D.J., Gainetdinov, R.R., Sotnikova, T.D., Zeng, H., Caron, M.G., and Tonegawa, S. (2003). Conditional calcineurin knockout mice exhibit multiple abnormal behaviors related to schizophrenia. Proc Natl Acad Sci U S A *100*, 8987-8992.

Miyakawa, T., Yamada, M., Duttaroy, A., and Wess, J. (2001). Hyperactivity and intact hippocampus-dependent learning in mice lacking the M1 muscarinic acetylcholine receptor. J Neurosci *21*, 5239-5250.

Robertson, E.J. (1987). Embryo-derived stem cell lines. In Teratocarcinomas and embryonic stem cells: A practical approach, E.J. Robertson, ed. (Oxford, IRL Press), pp. 71-112.

Steru, L., Chermat, R., Thierry, B., and Simon, P. (1985). The tail suspension test: a new method for screening antidepressants in mice. Psychopharmacology (Berl) *85*, 367-370.

Takao, K., and Miyakawa, T. (2006). Light/dark transition test for mice. J Vis Exp, 104.

Tujimura, A., Matsuki, M., Takao, K., Yamanishi, K., Miyakawa, T., and Hashimoto-Gotoh, T. (2008). Mice lacking the kf-1 gene exhibit increased anxiety- but not despair-like behavior. Front Behav Neurosci.

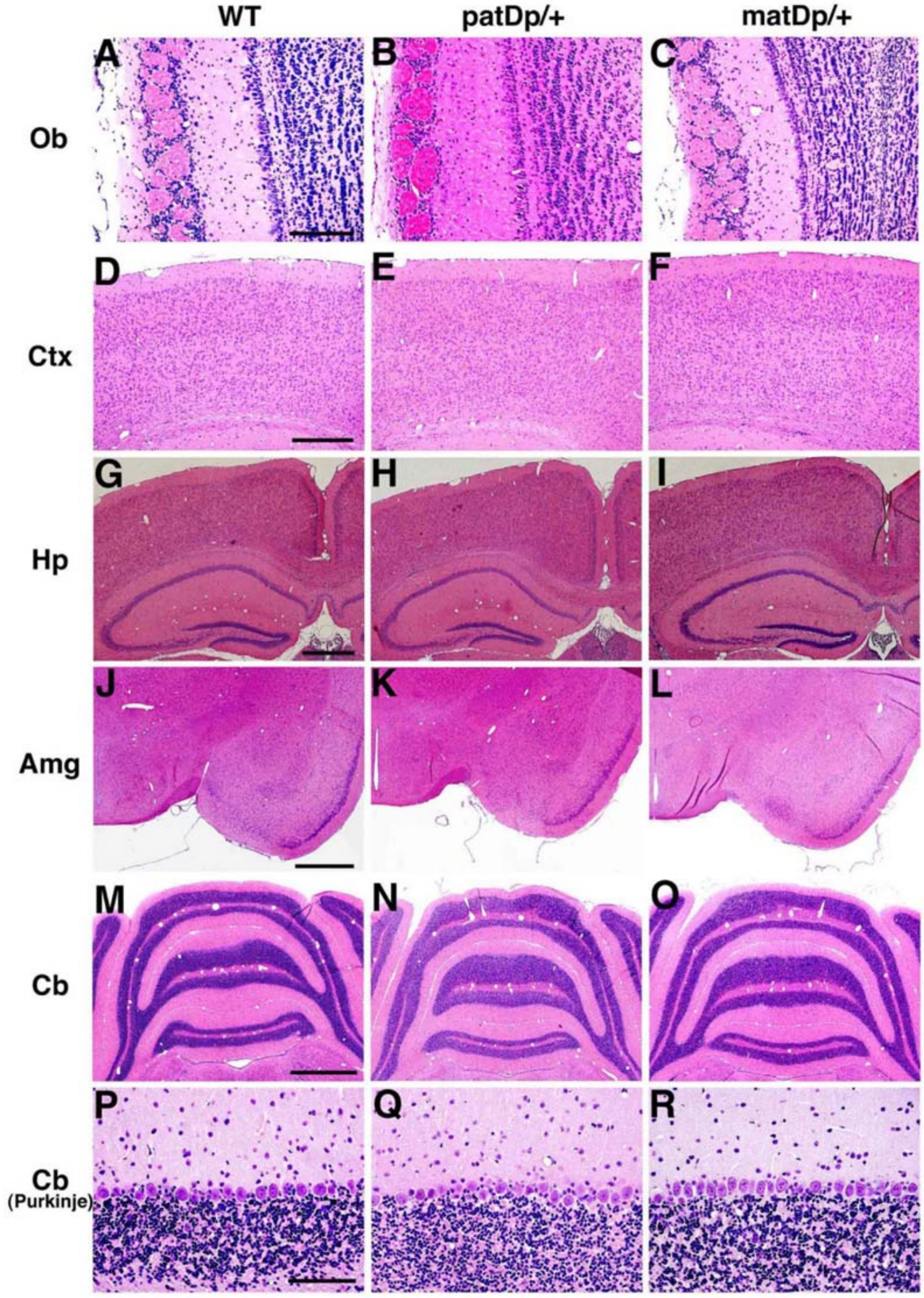

Figure S1. Histology of the adult mouse brain analysed by HE staining.
(**A** to **C**) Olfactory bulb (Ob), Scale bar, 200 µm. (**D** to **F**) Cortex (Ctx), Scale bar, 500 µm. The numbers of cortical cells in a 1.7 mm x 1.28 mm area of the retrosplenial cortex did not show any significant difference between *patDp*/+, *matDp*/+ and WT mice. (**G** to **I**) Hippocampus (Hp), Scale bar, 500 µm. (**J to L**) Amygdala (Amg), Scale bar, 1 mm. (**M** to **O**) Cerebellum (Cb), Scale bar, 1 mm. (**P** to **R**) Cb (Purkinje). Scale bar, 100 µm.

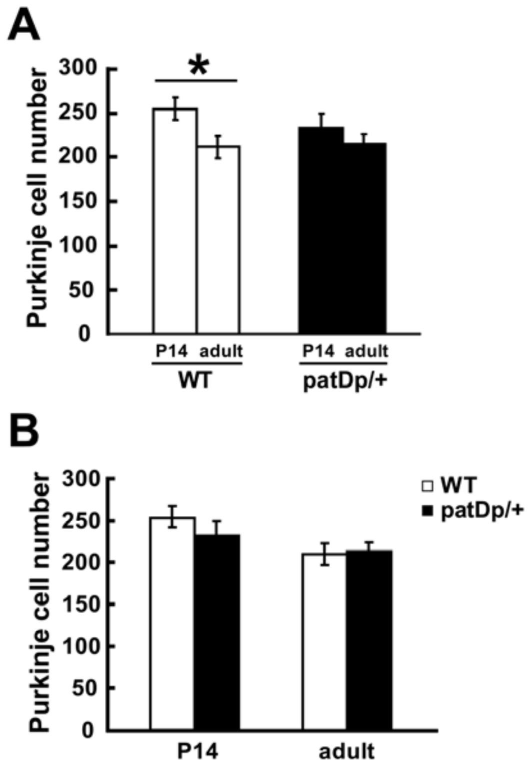

Figure S2. The number of Purkinje cells in the cerebellum.
The number of Purkinje cells in the cerebellar simple lobule was counted along a 500 µm length of the Purkinje-cell layer. P14 (n=3) and adult (n=3) mice were used. (**A**) In wild type mice, the number of cells at P14 was significantly greater compared with the adult mice. *, $p<0.05$; t-test. However, there was no significant difference in *pat*Dp/+ mice. (**B**) No significant difference between the two genotypes both at P14 and in the adult. Error bars indicate SEM (standard error of the mean).

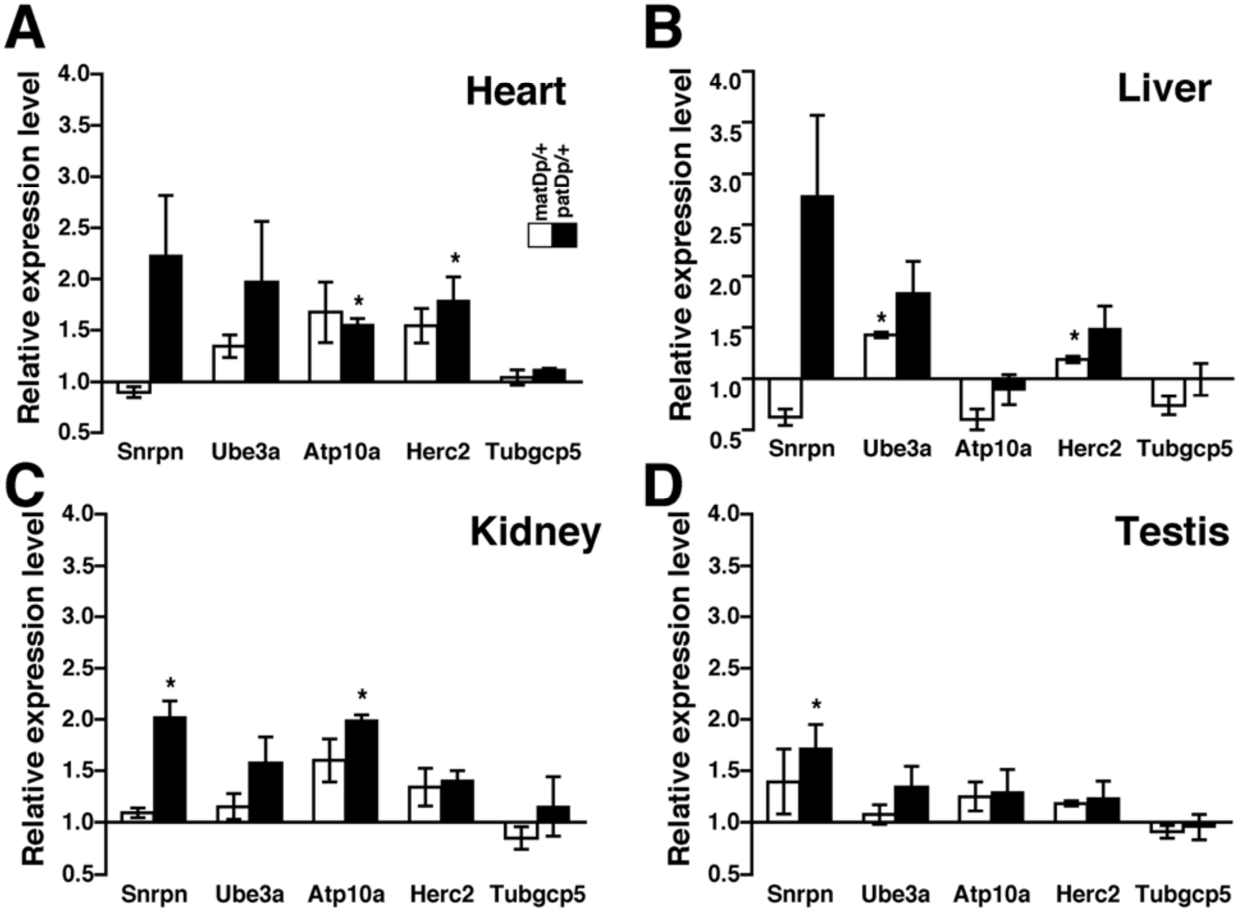

Figure S3. mRNA expression in mouse peripheral tissues analysed by quantitative RT-PCR. The expression levels of *patDp*/+ (n=3) and *matDp*/+ (n=3) were compared with that of WT (n=3), which was defined as 1.0. *Tubgcp5* is a control gene that is located outside the duplicated region. Error bars, SEM. *, $p<0.05$.

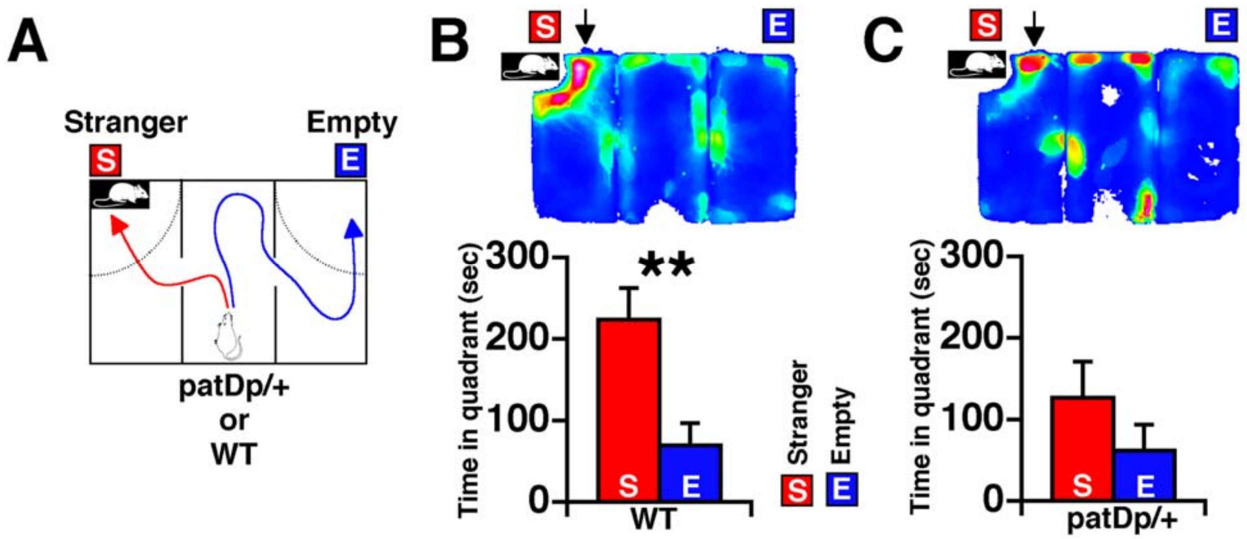

Figure S4. ***patDp/+*** **(129SvEv) mice have decreased sociability.**
Three-chamber test, (**A**) Schematic representaion of the 3-chambered apparatus. S and E represent the stranger mouse and empty, respectively. The quadrant spaces depicted by the dotted lines were used for quantitative analysis. Average image for all traces of WT (upper **B**, n=22) and *patDp*/+ mice (upper **C**, n=21). Comparison of time spent in the quadrant spaces between S and E for WT mice (lower **B**) and *patDp*/+ (lower **C**). Error bars, SEM. **, p<0.01. A stranger mouse is restricted in one of the side chambers in a wire cage (depicted as S), and the opposite chamber is left empty (E). Images of movement traces for 10 min (WT; n=22, *patDp/+*; n=21) are shown in B and C with the warmer colors representing more time spent in that particular location. (**B**) WT mice tended to contact the stranger mouse (arrow) and the time spent with the stranger mouse in the quadrant location depicted by the dotted line in A was significantly higher than that in the opposite empty chamber. (**C**) In contrast, the *patDp/+* mice tended to stay more in the central chamber, preferably in the corners. Some stayed longer at the edges (arrow). Thus, no significant difference in time spent between the quadrant spaces of either side was observed.

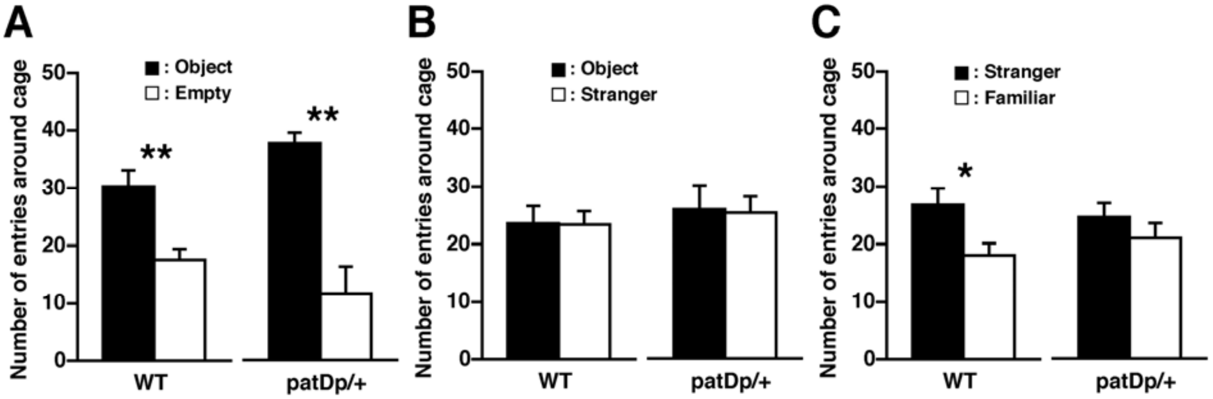

Figure S5. *patDp/+* mice show decreased sociability.
(**A**) A novel object (a dodecahedral pole) is placed in a cage in the chamber on one side and no object in the chamber on the other side. Both WT and *patDp/+* mice showed more number of entries around the cage with a novel object. **, p<0.001. (**B**) Another novel object (a cone) is placed in a cage in the chamber on one side and an adult conspecific mouse (C57BL/6J) that has had no previous contact with the subject (test mouse) is placed in a cage in the chamber on the other side. (**C**) A novel stranger mouse (C57BL/6J) is placed in a cage in the chamber on one side and a familiar mouse that has been used in a previous test in B is placed in the chamber on the other side. n=11. *, p=0.05. These data were evaluated by *t*-test. Error bars, SEM.

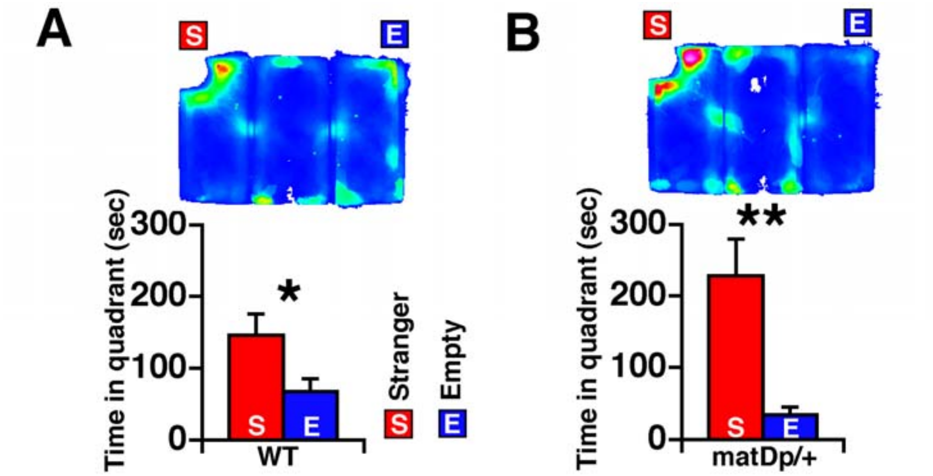

Figure S6. *matDp*/+ mice do not show any significantly different behavior compared with WT in the three-chamber test.
Average image for all traces of WT (upper A, n=18) and *matDp*/+ mice (upper B, n=18). Comparison of time spent in quadrant spaces between S and E in WT (lower A) and *matDp*/+ mice (lower B). Error bars, SEM. **, p<0.01. *, p<0.05.

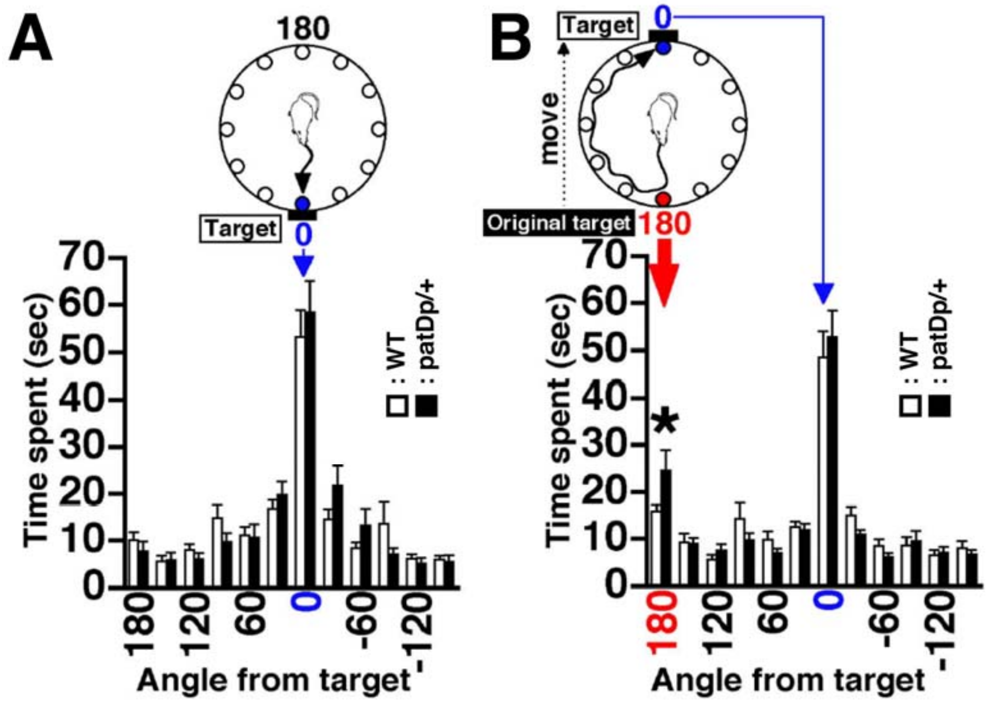

Figure S7. Behavioral inflexibility of *patDp/+* mice.
Barnes maze test, n=21; *patDp*/+, n=22; WT. (**A**) The hole at 0 degrees is the correct hole chosen as the target. **(B)** The reversal learning task of the Barnes maze. Error bars, SEM. *, $p<0.05$.

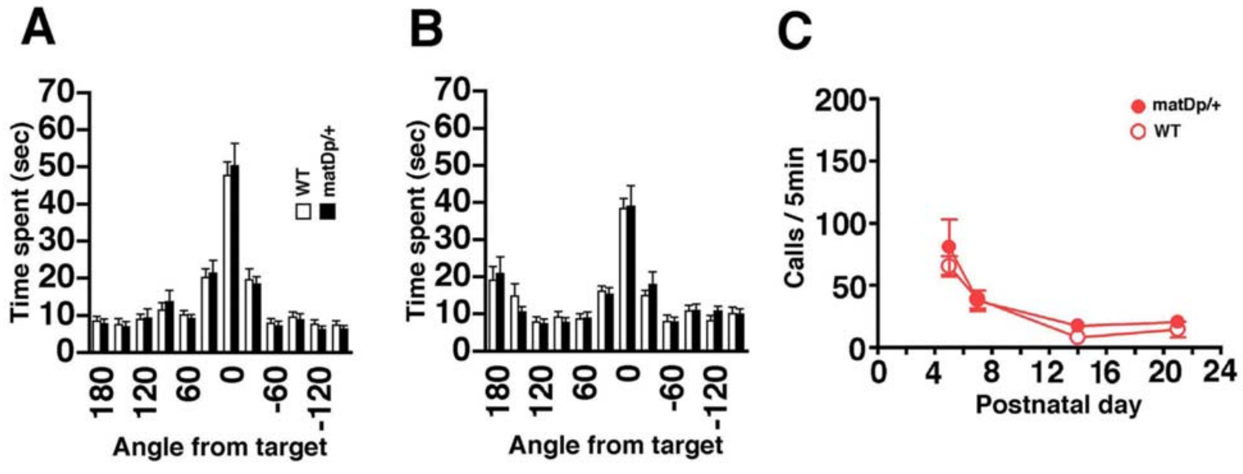

Figure S8. *matDp*/+ mice do not show any significantly different behavior compared with WT in the Barnes maze test and USVs.
(**A**) Barnes maze test, n=17; *matDp*/+ mice, n=17; WT mice. (**B**) The reversal learning task of the Barnes maze. Error bars, SEM. (**C**) Maternal separation-induced ultrasonic vocalizations at P5, 7, 14, and 21 (or 22). n=13, 17, 17, and 9; *matDp*/+ mice, n=16, 25, 25, and 12; WT mice. Error bars, SEM.

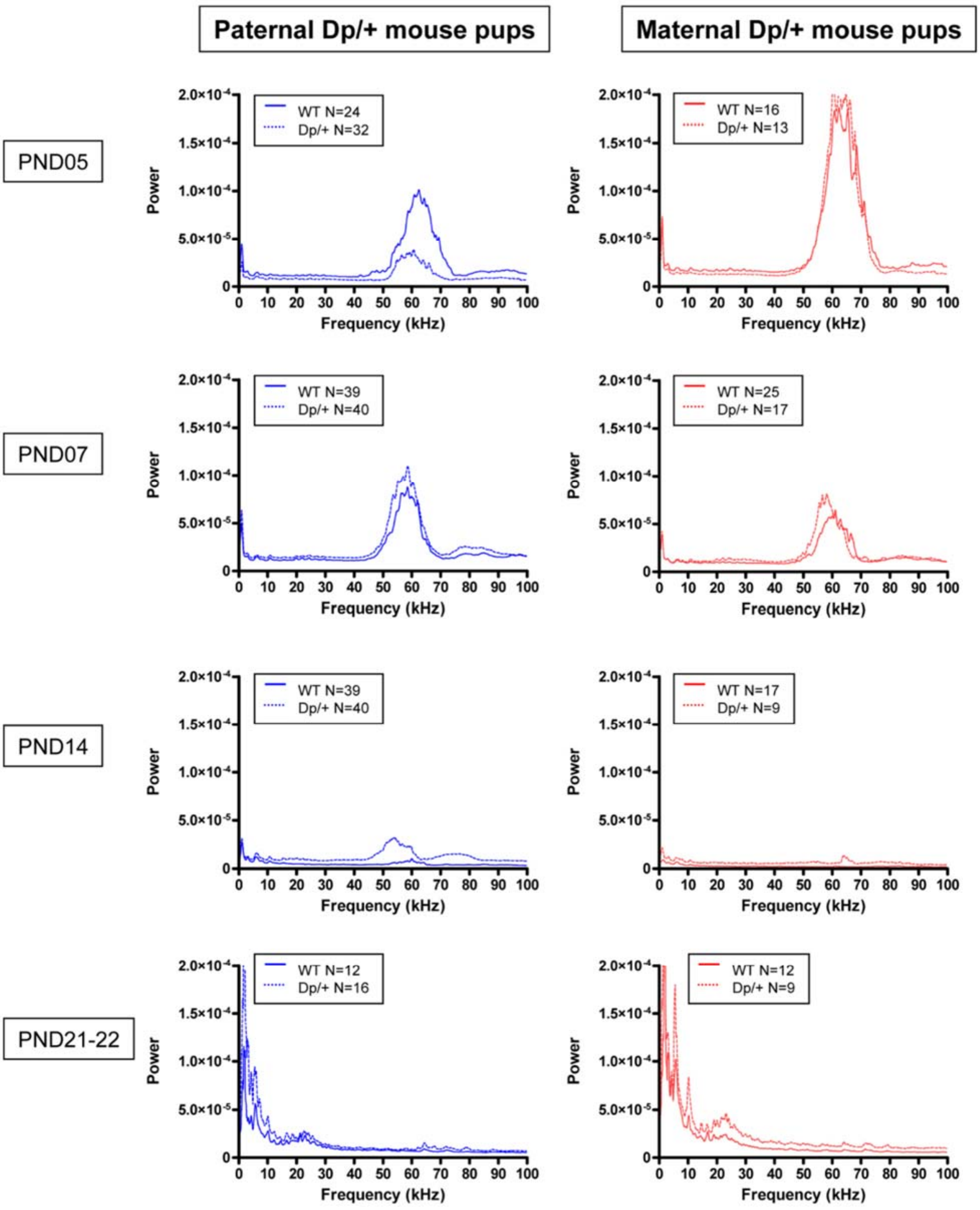

Figure S9. Changes in USV frequency distribution during postnatal periods in mouse pups with the duplication.
When the power spectrum of vocalization frequency was generated, a digital filter at 20 kHz was disengaged.

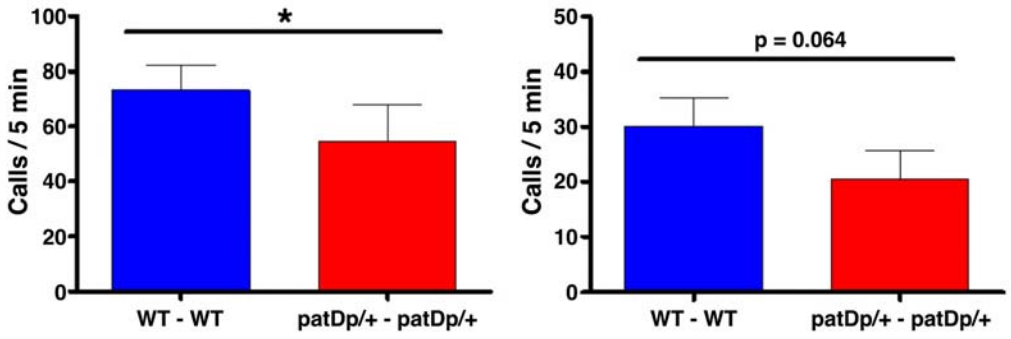

Figure S10. Adult *patDp/+* mice exhibit decreased vocalization in the resident-intruder test. (**left**) Total vocalization ranging from audible to ultrasonic bands during the last 5 min in a 10 min resident-intruder paradigm. (**right**) Ultrasonic vocalizations during the last 5 min in a 10 min resident-intruder paradigm. Seven pairs were analyzed for both WT and *patDp/+* mice. One-tailed Mann-Whitney U test was used to compare the difference in the number of vocalizations between pairs of WT and *patDp/+* mice. * p<0.05. Error bars, SEM.

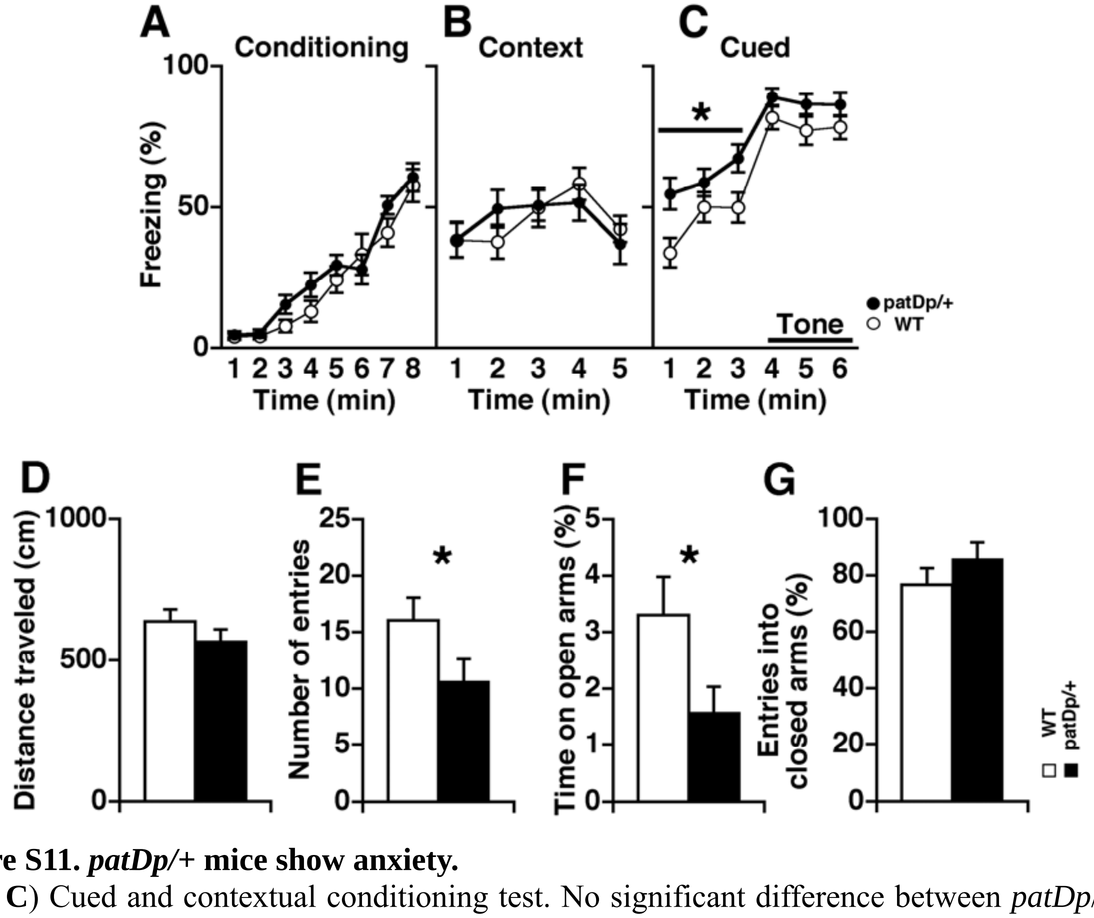

Figure S11. *patDp/+* mice show anxiety.
(**A** to **C**) Cued and contextual conditioning test. No significant difference between *patDp*/+ and WT mice during conditioning training (**A**) or in the same contextual condition (**B**). n=22 both for *patDp*/+ and WT mice. In the altered contextual environment (**C**), *patDp*/+ mice exhibited higher

freezing scores during the first 3 minutes in the absence of the cue compared with WT. Error bars, SEM. *, p<0.05. (**D** to **G**) Elevated plus maze test. n=22 both for *patDp*/+ and WT mice. No significant difference in traveled distance between *patDp*/+ and WT mice (**D**). The number of entries (**E**) and time on the open arms (**F**) for *patDp*/+ mice were significantly decreased compared with those for WT. Error bars, SEM. *, p<0.05. (**G**) There was no significant difference in entries into the closed arm between *patDp*/+ and WT mice.

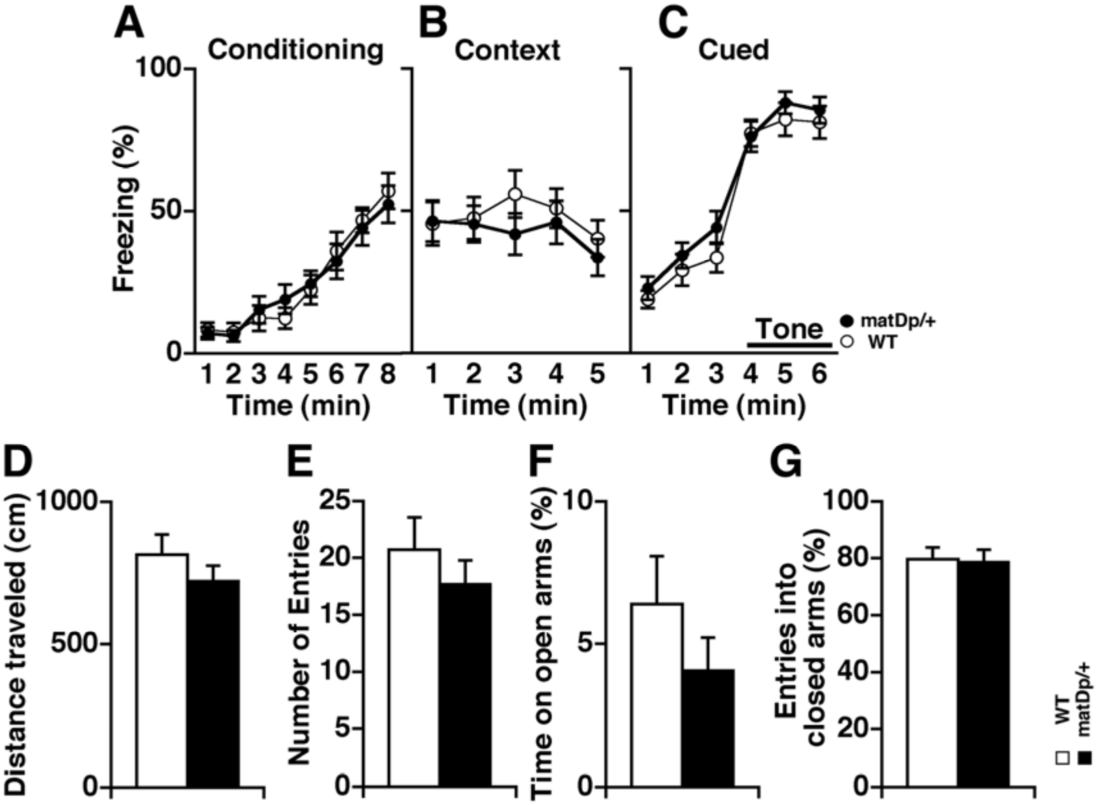

Figure S12. *matDp*/+ mice do not show anxiety.
(**A** to **C**) Cued and contextual conditioning test. n=18; *matDp*/+ mice, n=18; WT mice. There was no significant difference between *matDp*/+ and WT mice during conditioning training (**A**), in the same contextual condition (**B**) and in the altered contextual environment in the presence of the cue (**C**). Error bars, SEM. (**D** to **G**) Elevated plus maze test. n=18; *matDp*/+ mice, n=18; WT mice. There was no significant difference in traveled distance (**D**), the number of entries (**E**), time on open arms (**F**) entries into the closed arms (**G**) between *matDp*/+ and WT mice. Error bars, SEM.

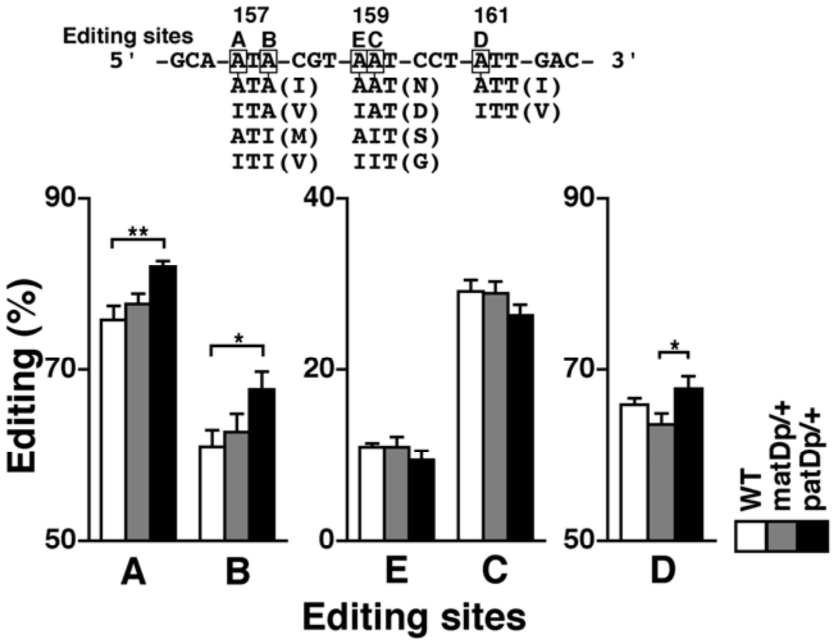

Figure S13. RNA editing sites on the 5-HT2cR and editing ratios.
Five boxed sites of adenine (A-E), their possible triplet codes (amino acids), and their encoded amino acid numbers (157, 159 and 161) are shown in the upper portion. Error bars, SEM. n=4; *patDp*/+ mice, n=5; *matDp*/+ mice, n=7; WT mice. **, p<0.01, *, p<0.05.

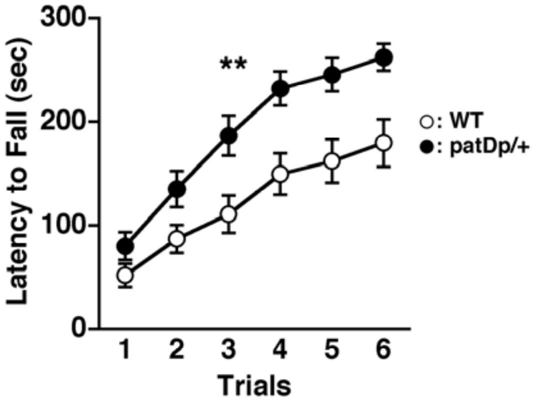

Figure S14. Rotarod performance of *patDp*/+ mice.
patDp/+ mice showed a significantly greater improvement in rotarod performance compared with WT mice. n=21; *patDp*/+ mice, n=22; WT mice. Error bars, SEM. **, p<0.01.

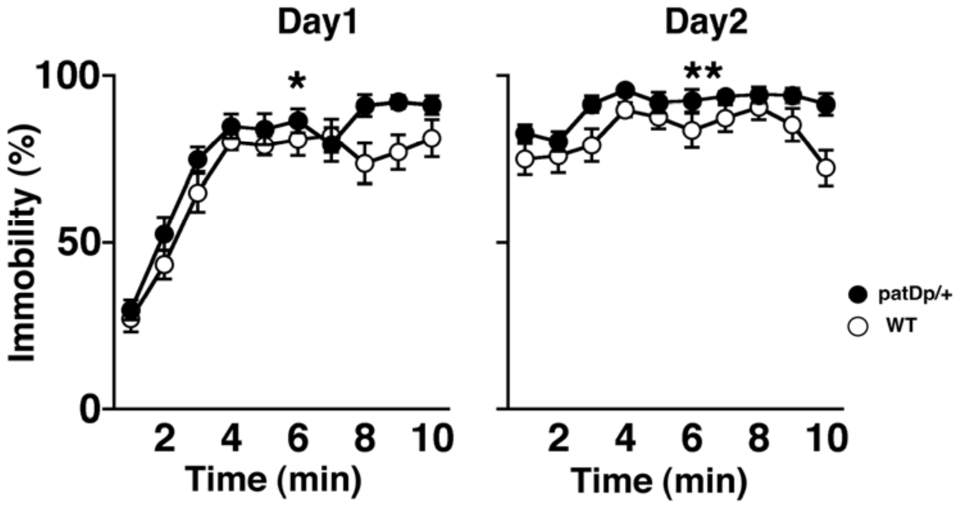

Figure S15. *patDp*/+ mice show anxiety.
Porsolt forced swim task. *patDp*/+ mice showed higher levels of immobility compared with WT mice both at Day 1 and Day 2. Error bars, SEM. n=22; *patDp*/+ mice, n=22; WT mice. *, p<0.05, **, p<0.01.

Table S1. A list of behavioral tests performed (WT vs. *patDp/+* mice)

Behavioral test			Mean ± SEM		*F* value	*P* value[a]
			WT	patDp/+		
General health	Weight (g)		25.309 ± 0.521	25.382 ± 0.588	F(1,42)=0.009	0.9267
	Temperature (°C)		36.614 ± 0.153	36.382 ± 0.123	F(1,42)=1.396	0.244
Pain test	Hot plate (latency, sec)		5.959 ± 0.524	6.614 ± 0.347	F(1,42)=1.084	0.3037
Motor tests	Grip strength (*N*)		0.872 ± 0.021	0.908 ± 0.023	F(1,42)=1.337	0.2542
	Wire hang (latency to fall, sec)		49.14 ± 3.861	41.395 ± 4.944	F(1,42)=1.524	0.2238
	Rotarod (latency to fall, sec; average of 6 trials)		123.317 ± 19.859	190.061 ± 20.992	F(1,41)=10.026	0.0029 *
Anxiety-like behavior	Light/dark transition	Distance travelled light (cm)	212.341 ± 36.634	194.718 ± 41.553	F(1,42)=0.101	0.752
		Distance travelled dark (cm)	637.855 ± 72.399	665.655 ± 58.686	F(1,42)=0.089	0.7669
		Stay Time in light (sec)	131.159 ± 29.395	86.773 ± 17.605	F(1,42)=1.678	0.2023
		Transitions (times)	8.5 ± 2.154	8.818 ± 2.1	F(1,42)=0.011	0.9163
		Latency to light (sec)	240.409 ± 50.645	252.182 ± 56.099	F(1,42)=0.024	0.877
	Elevated plus maze	Number of entries (times)	16.045 ± 1.987	10.636 ± 1.482	F(1,42)=4.76	0.0348 *
		Distance travelled (cm)	635.923 ± 42.879	563.991 ± 44.283	F(1,42)=1.362	0.2498
		Entries into open arms (%)	18.815 ± 4.611	14.149 ± 4.227	F(1,42)=0.556	0.4599

		Time on open arms (%)	3.303 ± 0.673	1.557 ± 0.474	F(1,42)=4.492	0.04 *
		Time on closed arms (%)	41.83 ± 5.608	39.951 ± 6.767	F(1,42)=0.046	0.8318
		Time on center arms (%)	54.867 ± 5.391	58.492 ± 6.543	F(1,42)=0.183	0.6711
Depression model	Porsolt forced swim (immobility, %)	Day1	68.960 ± 4.026	76.659 ± 4.304	F(1,42)=5.907	0.0194 *
		Day2	82.703 ± 1.407	90.813 ± 1.086	F(1,42)=8.971	0.0046 *
	Tail suspension (immobility, %)		38.876 ± 2.632	40.365 ± 3.445	F(1,42)=0.065	0.7999
Locomotor activity	Open field	Total distance (cm)	17314.917 ± 1175.132	21645.462 ± 2035.514	F(1,42)=0.262	0.6111
		Vertical activity (times)	1253.25 ± 92.566	1586.846 ± 163.34	F(1,42)=0.881	0.3534
		Center time (sec/min)	8.642 ± 1.329	5.711 ± 0.854	F(1,42)=0.697	0.4084
		Stereotypic count (times)	11198.5 ± 447.585	11506 ± 750.506	F(1,42)=0.532	0.47
Social interaction (one-chamber)	Total duration of contact (sec)		133.545 ± 30.317	222.291 ± 51.266	F(1,20)=2.220	0.1518
	Number of contacts (times)		32.636 ± 5.035	25.455 ± 4.057	F(1,20)=1.234	0.2799
	Total duration of active contacts (sec)		8.764 ± 1.322	5.491 ± 0.836	F(1,20)=4.377	0.0494 *
	Mean duration/Contact		4.136 ± 1.052	19.255 ± 9.236	F(1,20)=2.645	0.1195
	Distance travelled (cm)		2837.818 ± 396.169	2061.455 ± 282.966	F(1,20)=2.543	0.1265
Sensory motor gating	Acoustic startle response	110-dB	0.928 ± 0.187	1.573 ± 0.18	F(1,42)=6.155	0.0172 *
		120-dB	3.593 ± 0.343	3.583 ± 0.349	F(1,42)=0.000381	0.9845

	Prepulse inhibition (startle stimulus, %)	74-110-dB	43.966 ± 7.139	55.17 ± 3.978	F(1,42)=1.869	0.1788
		78-110-dB	48.764 ± 7.537	64.539 ± 4.103	F(1,42)=3.379	0.0731
		74-120-dB	22.549 ± 3.997	21.345 ± 5.031	F(1,42)=0.035	0.8522
		78-120-dB	37.152 ± 4.499	31.314 ± 4.708	F(1,42)=0.804	0.3751
Contextual and cued fear conditioning	Conditioning (freezing, %)		23.190 ± 5.818	27.090 ± 5.388	F(1,42)=1.102	0.2997
	Context (freezing, %)		45.201 ± 6.495	45.349 ± 5.966	F(1,42)=0.001	0.981
	Cue 1-6 min (freezing, %)		61.715 ± 6.158	73.698 ± 5.260	F(1,42)=6.404	0.0152 *
	Cue 1-3 min (freezing, %)		44.468 ± 3.147	60.155 ± 2.970	F(1,42)=6.494	0.0146 *
	Cue 4-6 min (freezing, %)		78.962 ± 4.388	87.241 ± 3.524	F(1,42)=2.958	0.0928

a *<0.05

Behavioral test battery was performed in the following order

General health/neurological screen, wire hang, grip strength test, light/dark transition, open field, elevated plus maze, hot plate, one-chamber social interaction, rotarod, prepulse inhibition, Porsolt forced swim, contextual and cued fear conditioning, three-chamber social interaction test, eight arm radial maze, barnes maze, tail suspension test, 24 hour home cage monitoring.

Table S2. A list of behavioral tests performed (WT vs. *matDp/+* mice)

Behavioral test			Mean ± SEM		*F* value	*P* value[a]
			WT	matDp/+		
General health	Weight (g)		24.733 ± 0.539	23.783 ± 0.54	F(1,34)=1.551	0.2215
	Temperature (°C)		37.039 ± 0.125	36.35 ± 0.114	F(1,34)=16.563	0.0003 *
Pain test	Hot plate (latency, sec)		5.9 ± 0.467	6.267 ± 0.588	F(1,34)=0.239	0.6283
Motor tests	Grip strength (*N*)		0.818 ± 0.032	0.803 ± 0.03	F(1,34)=0.118	0.7334
	Wire hang (latency to fall, sec)		57.548 ± 1.555	47.845 ± 4.877	F(1,34)=3.594	0.0665
	Rotarod (latency to fall, sec; average of 6 trials)		165.972 ± 24.433	209.194 ± 20.805	F(1,34)=2.929	0.0961
Anxiety-like behavior	Light/dark transition	Distance travelled light (cm)	458.394 ± 76.262	335.794 ± 60.666	F(1,30)=1.583	0.2181
		Distance travelled dark (cm)	829.781 ± 102.749	683.087 ± 85.098	F(1,30)=1.209	0.2803
		Stay Time in light (sec)	249.094 ± 40.492	163 ± 37.933	F(1,30)=2.408	0.1312
		Transitions (times)	16 ± 3.482	8.125 ± 2.655	F(1,30)=3.234	0.0822
		Latency to light (sec)	137.25 ± 45.996	220.062 ± 57.09	F(1,30)=1.276	0.2676
	Elevated plus maze	Number of entries (times)	20.722 ± 2.825	17.722 ± 2.045	F(1,34)=0.74	0.3957
		Distance travelled (cm)	814.161 ± 71.072	720.333 ± 54.738	F(1,34)=1.094	0.303
		Entries into open arms (%)	20.403 ± 4.16	21.121 ± 4.129	F(1,34)=0.015	0.9033
		Time on open arms (%)	6.389 ± 1.683	4.042 ± 1.168	F(1,34)=1.312	0.26

		Time on closed arms (%)	36.926 ± 5.744	33.19 ± 5.482	F(1,34)=0.221	0.641
		Time on center arms (%)	56.685 ± 5.751	62.769 ± 5.401	F(1,34)=0.595	0.446
Depression model	Porsolt forced swimming (immobility, %)	Day1	55.803 ± 9.307	65.973 ± 12.187	F(1,34)=0.297	0.5896
		Day2	72.002 ± 5.980	73.129 ± 6.512	F(1,34)=0.057	0.8129
	Tail suspension (immobility, %)		39.411 ± 6.613	42.774 ± 7.292	F(1,32)=0.344	0.5619
Locomotor activity	Open field	Total distance (cm)	17314.917 ± 1175.132	21645.462 ± 2035.514	F(1,34)=0.813	0.3737
		Vertical activity (times)	1253.250 ± 92.566	1586.846 ± 163.340	F(1,34)=1.273	0.2671
		Center time (sec/min)	8.642 ± 1.329	5.711 ± 0.854	F(1,34)=1.709	0.1999
		Stereotypic count (times)	11198.500 ± 447.585	11506.000 ± 750.506	F(1,34)=2.579	0.1175
Social interaction (one-chamber)	Total duration of contact (sec)		101.725 ± 26.355	144.978 ± 32.605	F(1,15)=1.030	0.3264
	Number of contacts (times)		34.5 ± 5.134	39.889 ± 5.891	F(1,15)=0.464	0.5061
	Total duration of active contacts (sec)		8.825 ± 1,288	9.111 ± 1.333	F(1,15)=0.024	0.8801
	Mean duration/Contact		2.988 ± 0.441	4.522 ± 1.466	F(1,15)=0.903	0.357
	Distance travelled (cm)		3432.125 ± 412.273	3302.889 ± 388.645	F(1,15)=0.052	0.8226
Sensory motor gating	Acoustic startle response	110-dB	1.023 ± 0.14	2.049 ± 0.448	F(1,34)=4.780	0.0358 *
		120-dB	2.749 ± 0.317	4.668 ± 0.605	F(1,34)=7.900	0.0081 *
	Prepulse inhibition (startle stimulus, %)	74-110-dB	57.249 ± 5.899	41.232 ± 9.067	F(1,34)=2.193	0.1479

		78-110-dB	64.072 ± 6.777	62.357 ± 4.512	F(1,34)=0.044	0.8344
		74-120-dB	19.41 ± 6.097	14.374 ± 4.685	F(1,34)=0.429	0.5169
		78-120-dB	35.054 ± 5.625	23.452 ± 4.944	F(1,34)=2.400	0.1306
Contextual amd cued fear conditioning	Conditioning (freezing, %)		25.290 ± 6.266	25.0674 ± 6.111	F(1,34)=0.002	0.9655
	Context (freezing, %)		47.941 ± 7.304	42.576 ± 6.922	F(1,34)=0.407	0.5279
	Cue 1-6 min (freezing, %)		53.672 ± 8.071	58.410 ± 7.638	F(1,34)=0.822	0.371
	Cue 1-3 min (freezing, %)		27.191 ± 2.816	33.702 ± 2.971	F(1,34)=1.432	0.2396
	Cue 4-6 min (freezing, %)		80.154 ± 5.323	83.119 ± 4.710	F(1,34)=0.211	0.649

a *<0.05
